# Incidence trends for twelve cancers in younger adults—a rapid review

**DOI:** 10.1038/s41416-022-01704-x

**Published:** 2022-02-07

**Authors:** Erica di Martino, Lesley Smith, Stephen H. Bradley, Scott Hemphill, Judy Wright, Cristina Renzi, Rebecca Bergin, Jon Emery, Richard D. Neal

**Affiliations:** 1 Division of Primary Care, Public Health & Palliative Care, Leeds Institute of Health Sciences, University of Leeds, Worsley Building, Clarendon Way, Leeds, LS2 9NL UK; 2 Leeds Centre for Personalised Medicine and Health, University of Leeds, Worsley Building, Clarendon Way, Leeds, LS2 9NL UK; 3 Academic Unit of Health Economics, Leeds Institute of Health Sciences, University of Leeds, Worsley Building, Clarendon Way, Leeds, LS2 9NL UK; 4 Epidemiology of Cancer Healthcare and Outcomes Research Group, Department of Behavioural Science and Health, Institute of Epidemiology & Health Care, University College London, London, UK; 5 Centre for Cancer Research, Faculty of Medicine, Dentistry and Health Sciences, University of Melbourne, Victoria Comprehensive Cancer Centre, 305 Grattan Street, Melbourne, VIC 3000 Australia; 6 Cancer Epidemiology Division, Cancer Council Victoria, 615 St Kilda Rd, Melbourne, VIC 3002 Australia

**Keywords:** Cancer epidemiology, Cancer epidemiology

## Abstract

Many cancer referral guidelines use patient’s age as a key criterium to decide who should be referred urgently. A recent rise in the incidence of colorectal cancer in younger adults has been described in high-income countries worldwide. Information on other cancers is more limited. The aim of this rapid review was to determine whether other cancers are also increasing in younger age groups, as this may have important implications for prioritising patients for investigation and referral. We searched MEDLINE, Embase and Web of Science for studies describing age-related incidence trends for colorectal, bladder, lung, oesophagus, pancreas, stomach, breast, ovarian, uterine, kidney and laryngeal cancer and myeloma. ‘Younger’ patients were defined based on NICE guidelines for cancer referral. Ninety-eight studies met the inclusion criteria. Findings show that the incidence of colorectal, breast, kidney, pancreas, uterine cancer is increasing in younger age groups, whilst the incidence of lung, laryngeal and bladder cancer is decreasing. Data for oesophageal, stomach, ovarian cancer and myeloma were inconclusive. Overall, this review provides evidence that some cancers are increasingly being diagnosed in younger age groups, although the mechanisms remain unclear. Cancer investigation and referral guidelines may need updating in light of these trends.

## Introduction

Growing evidence suggests that younger patients with cancer are more likely to experience a diagnostic delay. As cancer is more common in the elderly, doctors are more inclined to suspect cancer in older patients [1] and younger patients are more likely than older people to have consulted with a doctor three or more time before referral [2, 3]. Even when referred, younger patients may be referred through a less urgent route compared to older ones [4]. A delay in diagnosis may result in cancer progressing to a less curable stage. Some studies suggest that for some cancers younger patients have more advanced disease at diagnosis compared to older ones [5, 6].

Clinical guidelines for cancer referral are based on the positive predictive value of symptoms, which indicates the likelihood that a certain symptom or symptom’s combination may be caused by cancer. As cancer incidence increases with age, this positive predictive value is higher in older patients. Therefore, many clinical guidelines, such as the UK NICE guidelines for cancer recognition and referral (https://www.nice.org.uk/guidance/ng12), use age as a key criterion to determine which patients require urgent investigation for suspected cancer.

Several recent reports have suggested that colorectal cancer is becoming more common in younger patients. For example, a global study found an increase in the incidence of colorectal cancer in adults under 50 years of age in 19 out of the 36 countries examined, nine of which had a stable or declining pattern in older adults [7]. Although similar changes have been described in other types of cancer [8], the data are more limited and there is a lack of comprehensive reviews of the evidence. An increase in the incidence of cancer in younger patients may mean that the predictive value of symptoms may be higher than previously estimated. Referral guidelines may therefore require revision, to avoid younger people experiencing greater diagnostic delays and consequent later stage at diagnosis and less treatable disease.

The aim of this rapid review is to collate and summarise recent epidemiological data on the incidence trends of twelve types of cancer in younger adults to inform and underpin health policy and help address age-related inequalities in cancer diagnosis.

## Methods

### Search strategy

In August 2020, we searched Ovid MEDLINE(R) and Epub Ahead of Print, In-Process & Other Non-Indexed Citations and Daily 1946 to August 19, 2020, Embase Classic+Embase (Ovid) 1947 to 2020 August 20, Science Citation Index-Expanded (Web of Science) 1900-present, Social Sciences Citation Index (Web of Science) 1900-present and Emerging Sources Citation Index (Web of Science) 2015-present to identify studies describing trends in cancer incidence in younger patients. The search included subject headings, text-words, and their synonyms for the search concepts of the cancers of interest, young and middle-aged adults, and change in incidence or prevalence. The searches were limited to studies published from 1995 onwards. Non-English language reports, editorials, letters, and case reports (but not retractions) were removed. Searches were peer-reviewed by a second information specialist. Complete search strategies are listed in the Supplementary Methods. Additional studies were sought by manually checking the references of papers that were identified through the initial literature search. All records were stored in an EndNote™ library, where duplicates were removed, before transferring to Rayyan© software for abstract screening.

### Eligibility criteria

We included in the search the twelve adult cancers that have a minimum age threshold for investigation in the UK NICE cancer referral guidelines: colorectal, bladder, lung, oesophagus, pancreas, stomach, breast, ovarian, uterine, kidney, and laryngeal cancer and myeloma. ‘Younger cancer patients’ were defined as individuals diagnosed below the age threshold in the NICE guideline for the specific cancer under consideration (Supplementary Table 1). Paediatric patients (<18-year olds) were excluded.

Other inclusion criteria were: (1) Language: English; (2) Period: 1995–2020; (3) Setting: OECD country, to have comparable populations in terms of average age, risk factors and lifestyle; (4) Type of study: reporting incidence trend over time in ‘younger patients’ (as defined above) separate from other age groups; (5) Outcome: a quantitative measure of change in incidence over time such as annual percentage change (APC), Average APC (AAPC) or Estimated APC (EAPC), obtained from a modelling approach such as joinpoint regression or age–period–cohort models.

Exclusion criteria were: (1) studies on secondary cancers or metastasis; (2) studies looking only at cancers in limited patient subgroups, for example, those with specific cancer syndromes or immune disorders; (3) studies examining single histological or pathological subtypes; (4) studies reporting trends in graphical format only, as extrapolation or secondary analysis of data was beyond the scope of this review; (5) studies not including at least 5 years of data after 1995; (6) editorials, case-report, expert opinion, studies only published as abstract.

### Data collection

Titles and abstracts were screened independently by two authors to determine eligibility. Studies for which there was disagreement between reviewers were included for full-text screening. This was conducted by one of five of the study authors. Where a full-text article was excluded by an author, eligibility was assessed by another author and disagreements resolved through discussion. Reasons for exclusion were recorded. Data extraction was undertaken with a predesigned template (Supplementary Methods).

### Assessment of quality of evidence

The quality of the studies was assessed using the Joanna Briggs Checklist for prevalence studies (https://jbi.global/critical-appraisal-tools), assigning a score of one for each of the criteria met.

### Synthesis

Findings were summarised using a narrative synthesis and presented visually in the form of tables and figures for each cancer type. For colorectal and breast cancer, random-effects meta-analysis was used to derive pooled estimates of the APCs and forest plots for graphical visualisation of results. Pooled estimates were calculated for studies reporting trends for similar age groups if there were at least three studies per age group. Further details of this analysis are reported in Supplementary Methods. When a study reported an APC for more than one period, only the most recent trend was considered.

## Results

### Search results

**Fig. 1:**
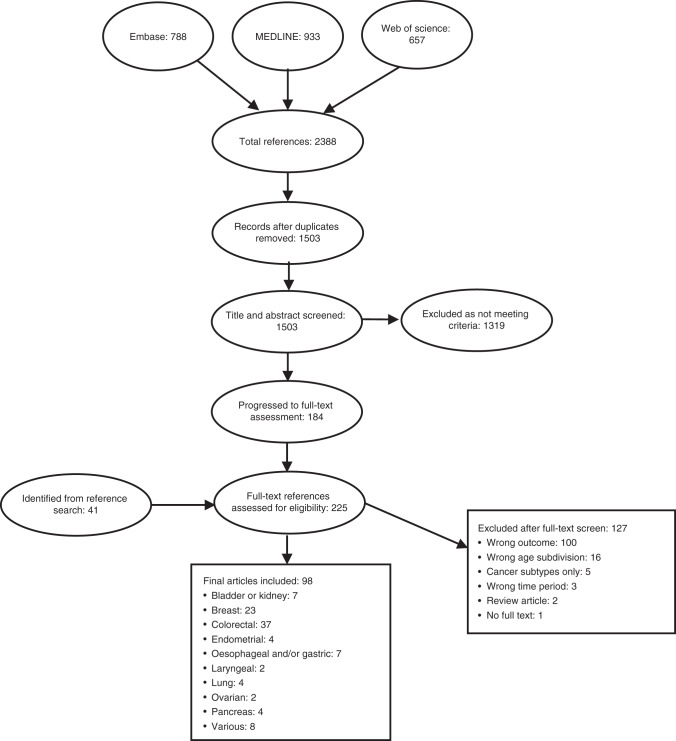
Review PRISMA flow diagram. The final number of articles included in the review was 98. A total of 2388 references were identified from Embase, MEDLINE and Web of Science. After the removal of duplicates, 1503 abstracts were assessed for eligibility. Of these, 225 were progressed to full-text assessment, along with other 41 publications identified through manual reference screening. After full-text review, 98 articles were identified as eligible, whilst 127 references were excluded.

We identified 1503 references through database searches and 41 from manual reference checks, with 225 progressing to the full-text screen. Ninety-eight studies satisfied all the inclusion criteria and were selected for the review (Fig. 1).

All studies had a retrospective design and were based on national or local cancer registries. Sixty-eight studies originated from North America, 24 from Europe, 3 from Oceania, 2 from Asia and 1 from South America. Five studies compared several countries worldwide.

Most studies contributed data for one type of cancer, but eight provided data for two or more. The most represented cancers were colorectal and breast. The quality of the studies was generally good, with a mean score of 8 (range 6–9), and 78 out of 98 studies scored 8 or above, out of the maximum possible score of 9. A list with details of the included studies is available in Supplementary Table 2.

### Cancer trends in younger adults

For some cancers, there was a clear and reproducible trend towards either an increase or a decrease in younger patients. For others, the studies included in the review reported conflicting results and no conclusion could be drawn on whether their incidence is changing, and in which direction. Based on this, we identified three distinct groups of cancers: those with consistent evidence of rising incidence in younger age groups (colorectal, breast, pancreas, kidney and uterine cancers), those with consistent evidence of decreasing incidence in younger age groups (bladder, lung and laryngeal cancer), and those for which the data were deemed inconclusive (stomach, oesophageal, ovarian cancer and myeloma) (Table 1). For the cancers with a clear trend towards an increase or a decrease, we considered the evidence ‘strong’ when coming from more than ten good-quality studies and ‘moderate’ when coming from ten or less good-quality studies.

**Table 1:** Overview of recent cancer incidence trends in younger adults.

Cancer type	Number of studies	Countries	Overall Incidence trend	Strength of evidence
Cancers with consistent evidence of a rise in younger adults				
Colorectal	43	International (5), US (24), Canada (5), Australia (2), Italy (2), England (2), Denmark (1), Sweden (1), Republic of Ireland (1)	Significant increase in aged <50 years, especially the youngest subgroups (i.e. aged <30 years), in both genders and both colon and rectal subtypes, with a declining trend in those aged >50 years. The trend is observed in most wealthy countries worldwide, with some geographical variation in Europe.	Strong
Breast	28	US (14), Canada (3), Netherlands (2), France (1), Germany (1), Spain (1), Portugal (1), Switzerland (1), Colombia (1), more than one European country (3)	Small but significant increase in women aged <50 years in a wide range of OECD countries.	Strong
Kidney	10	US (7), Canada (3)	General increase in all age groups in North America, with evidence of a greater increase in those aged <45 years.	Moderate
Uterus	9	US (4), Canada (2), England (1), Norway (1), New Zealand (1)	Significant increase in women of all ages but evidence of a more pronounced rise in those aged 30–50 years in some countries.	Moderate
Pancreas	9	US (7), Canada (2)	Recent increase in those aged <40 years in North America, with some evidence of a more pronounced increase in women than in men.	Moderate
Cancers with a consistent evidence of decline in younger adults				
Lung	9	US (6), Canada (2), Spain (1)	Decrease in all age groups, but especially in younger ages (i.e. 30–50 years of age) and more pronounced in men than women.	Moderate
Bladder	6	US (4), Canada (2)	General decrease in all age groups with evidence of a steeper decrease in those aged <45 years.	Moderate
Laryngeal	6	US (2), Canada (2), the Netherlands (2)	General decrease, with evidence of a steeper decrease in younger age groups (<50 years of age).	Moderate
Cancers with inconclusive evidence				
Stomach	11	US (8), Canada (2), South Korea (1)	Decrease in those aged >50 years; conflicting evidence for those aged <50 years with a possible increase in the non-cardia adenocarcinoma subtype.	Inconclusive
Oesophagus	9	US (6), Canada (2), France (1)	Conflicting evidence but possible increase in adenocarcinomas and decrease in squamous cell carcinomas in those aged <50.	Inconclusive
Ovarian	7	US (3), Canada (2), South Korea (1), International (1)	Decrease in women aged >40 years; either stable or decreasing trend in younger women in most OECD countries, but an increase in Asia.	Inconclusive
Myeloma	4	US (2), Canada (2)	Some evidence of increase in all ages, but particularly patients aged <50 years in US; contrasting evidence in Canada.	Inconclusive

Cancer type, the total number of studies contributing evidence for each cancer, the countries where the studies were based and overall trend is reported. Cancers were divided based on whether the studies included in the review demonstrated a clear and consistent trend towards an increase or decrease in incidence. The strength of the evidence was considered ‘strong’ when coming from a large number of good-quality studies (>10) and ‘moderate’ when coming from a smaller number of good-quality studies (10 ore less).

### Cancers with evidence of an increase in younger age groups

#### Colorectal cancer

**Fig. 2:**
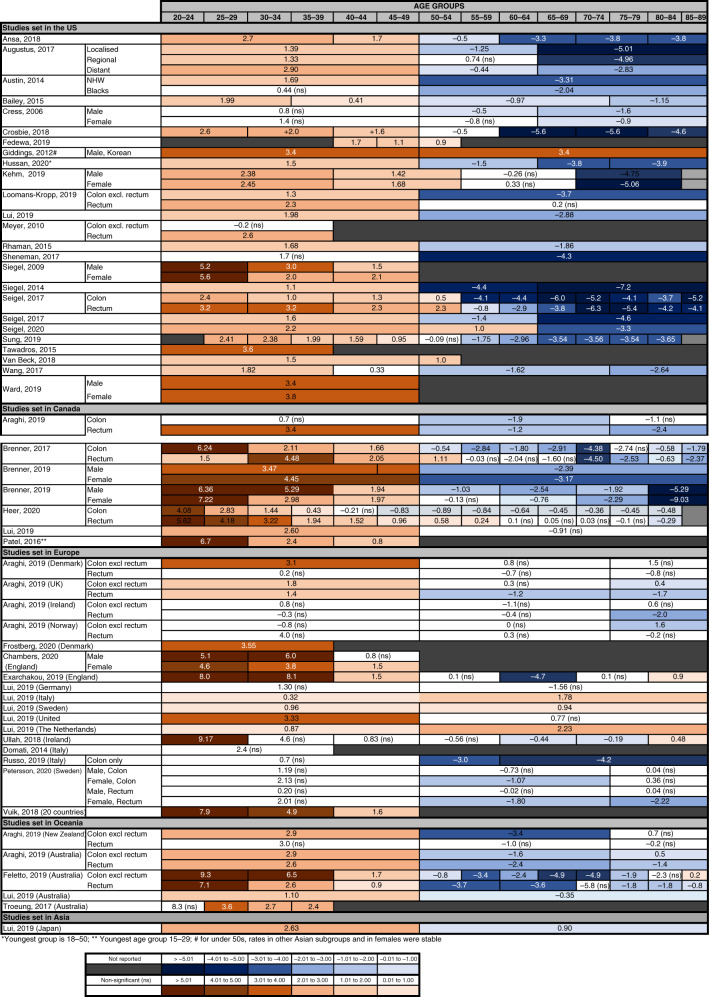
Recent trends in the incidence of colorectal cancer. Annual percentage changes (APC) in incidence are reported by age group. Increases are indicated in red and decreases in blue, with darker colours corresponding to greater changes. Stable incidences are indicated in white. For simplicity, the unstratified APC is reported, when available. For studies where the unstratified APC was not available, APC is stratified by anatomical location, histological type, gender and/or two main ethnicities (Black and NHW, non-Hispanics White). When APC values were available for several time periods, only the most recent APC is included. For some studies, the upper age limit for the oldest group and the lowest age limit for the younger group were not reported in the original study. Detailed information of the time periods and age groups covered by the different studies is reported in Supplementary Table 2. The following references are not included in the figure as they cover a large number of countries: Lu et al. [9] (20 countries worldwide), Siegel et al. [7] (36 countries worldwide).

Forty-three studies reported trends in the incidence of colorectal cancer in patients aged under 50 years (Table 1 and Fig. 2). Four of them examined international databases and described an increase in incidence in this age group in a wide range of wealthy countries worldwide, often accompanied by a decreasing incidence in those aged 50 and over [7, 9–11].

Twenty-nine studies looked at colorectal cancer incidence in North America [7–39] and all except two [16, 34] found a significant increase in the under 50s, of generally comparable magnitude in both men and women, with a corresponding decrease in older-age groups. APCs in younger patients were in the ranges of 1–3% when these were considered as a single group. When further subdivided by age and gender, the largest increase was observed in the youngest subgroups [8, 12, 15, 23, 28, 30, 33]. This was particularly so in Canada, where APCs of 6–7% were reported in under 30s [35–39], which are larger than the rise observed in the US in the same age group. One US study [24] found a stable rate of colon cancer in younger patients, but a rising rate of rectal cancer in the same age group. Data from other studies [14, 17, 20, 28–30] also suggested that the increase in colorectal cancer in the US may be driven predominantly by rectal tumours. In Canada, however, inconsistent results were found regarding anatomical location, with some studies showing a larger increase in colon and others in rectal cancers.

In Europe, large increases are observed in the younger population, with an annual rise between 2004 and 2016 of 7.9% in 20–29-year olds, 4.9% in 30–39-year olds and 1.6% in 40–49-year olds [40]. Some heterogeneity is noticeable between European countries with clear evidence of a rise in the under 50s in the UK [7, 9–11, 40–42] and Denmark [7, 9, 11, 40, 43] but a stable incidence in other countries.

In Australia, a similar increase in under 50s, especially the younger subgroups, and a corresponding decrease in over 50s was reported [7, 9–11, 44, 45]. In one study [45], the increase in under 50s was only statistically significant in women. Data from New Zealand were less consistent. Whilst an increased incidence of colorectal cancer in younger patients (APC 2.9–4.0) was observed in two international comparisons [7, 11], a third study [9] recorded no changes in young males and a small decrease of colon cancer in young females, together with a small increase in rectal cancer. We found one study including data from Asia [10], confirming a decrease in incidence in over 50 and an increase in under 50 in Japan.

Meta-analysis of studies with similar age group subdivision confirms an overall increase in patients below 50 years of age (pooled APC: 1.57, CI: 1.08–2.06), especially those in younger subgroups (pooled APC 20–29-year olds: 6.24, 95% CI: 4.79–7.69; pooled APC 30–39-year olds: 4.27, 95% CI: 2.98–5.56) (Supplementary Table 3 and Supplementary Fig. 2a–c).

Noticeably, some of the studies suggest that younger colorectal cancer patients often present with more aggressive diseases than older ones [23, 25, 43] and that the recent increase in incidence in younger patients is driven by more invasive cancers [13–15, 31–34, 46, 47]. For example, one study [47] reported that the proportion of patients under 50 presenting with Stage 4 colorectal cancer in the Republic of Ireland has doubled in recent years, from 11% in 1994 to 23% in 2012.

#### Breast cancer

**Fig. 3:**
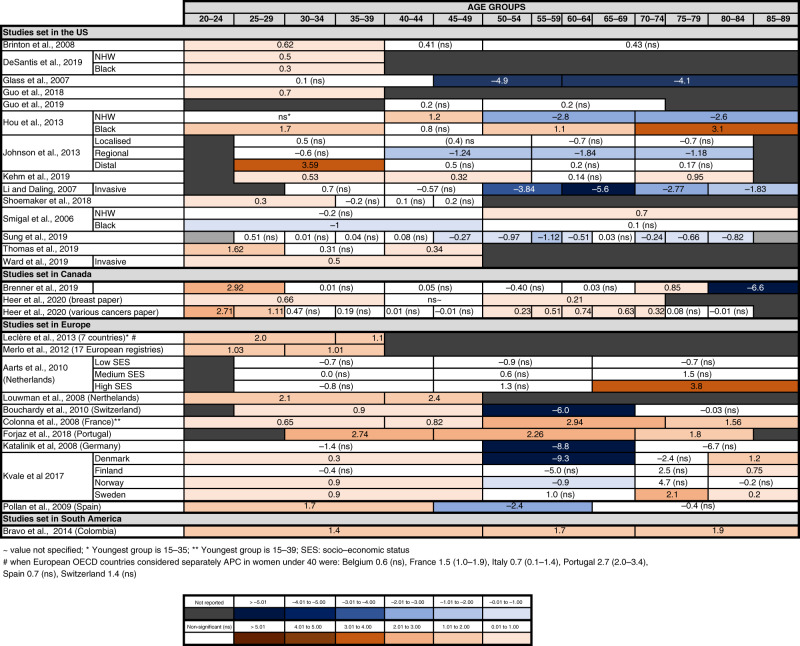
Recent trends in the incidence of breast cancer. Annual percentage changes (APC) in incidence are reported by age group. Increases are indicated in red and decreases in blue, with darker colours corresponding to greater changes. Stable incidences are indicated in white. For simplicity, the unstratified APC is reported, when available. For studies where the unstratified APC was not available, APC is stratified by gender and/or two main ethnicities (Black and NHW, non-Hispanics White). When APC values were available for several time periods, only the most recent APC is included. For some studies, the upper age limit for the oldest group and the lowest age limit for the younger group were not reported in the original study. Detailed information of the time periods and age groups covered by the different studies is reported in Supplementary Table 2.

We identified 28 publications reporting incidence trends of breast cancer in younger women (Table 1 and Fig. 3) [8, 26, 27, 37, 39, 48–70].

Almost all North-American studies found a recent increase in incidence in women under 50 [26, 27, 37, 39, 48–50, 53–56, 59], with only four studies reporting no change [8, 51, 52, 57] and one reporting a decrease in women of black ethnicity [58]. The magnitude of the change was modest, generally in the order of 0.5–1% per year. In some studies, the increase was limited to or more pronounced in the youngest subgroups [37, 39, 48, 49, 54, 59]. Several studies reported a significant decrease in older women (i.e. over 50) [8, 51, 55–57]. Some studies suggested that the observed increase in breast cancer in younger women in North America may be driven by invasive tumours [26, 27, 56]. For example, Johnson et al. reported no change in localised and regional disease but a strong increase in distal disease in women under 40 [56].

Eight out of the ten European studies also reported an increase in incidence in women under 50 [61–63, 65–69] with two finding no statistical change [60, 64]. In Portugal, the magnitude of change was larger than observed in the US and Canada, with APC around 2.7 [63, 66], but for the other countries the observed increase was ~1–2% per year.

The single South American study reports a similar increased risk of breast cancer in under 50 s in Colombia, as well as older-age groups [70].

Meta-analysis of studies with similar age group subdivision showed a small but significant increase in patients below 50 years of age (pooled APC: 0.94, 95% CI: 0.06–1.82). The larger increase was found in women aged 20–29 years (pooled APC: 2.45, 95% CI: 1.41–3.49) (Supplementary Table 3 and Supplementary Fig. 3).

#### Kidney cancer

**Fig. 4:**
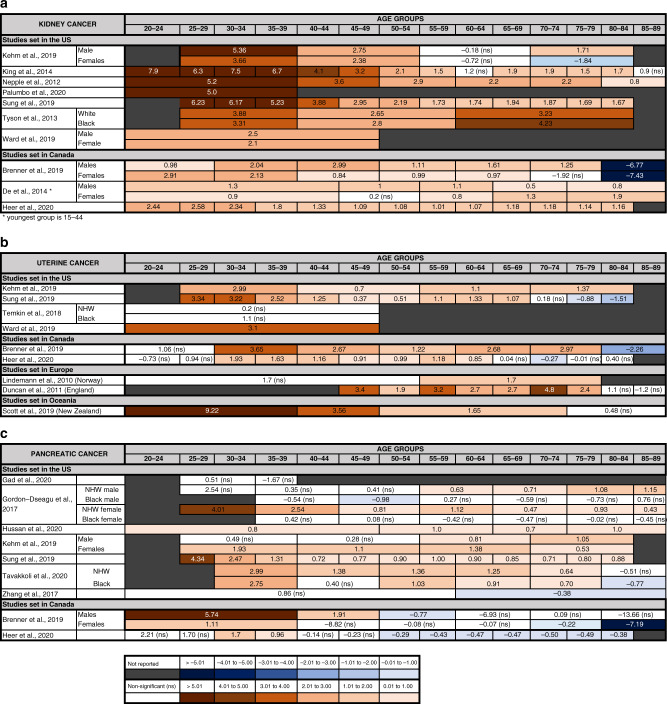
Other cancers with increasing incident trend in younger adults. Annual percentage changes (APC) in incidence of kidney (**a**), uterine (**b**) and pancreatic (**c**) cancer are reported by age group. Increases are indicated in red and decreases in blue, with darker colours corresponding to greater changes. Stable incidences are indicated in white. For simplicity, the unstratified APC is reported, when available. For studies where the unstratified APC was not available, APC is stratified by gender and/or two main ethnicities (Black and NHW, non-Hispanics White). When APC values were available for various time periods, only the most recent APC is included. For some studies, the upper age limit for the oldest group and the lowest age limit for the younger group were not reported in the original study. Detailed information of the time periods and age groups covered by the different studies is reported in Supplementary Table 2.

For kidney cancer, we found ten eligible studies, all from North America (Table 1 and Fig. 4a) [8, 26, 27, 37, 39, 71–75]. They consistently show a recent increase in the incidence of kidney cancer in all ages and in both genders, with six detecting a steeper rise in younger compared with older-age groups [8, 26, 37, 39, 71, 72]. The annual increase in patients under 45 varied between 0.9 and 7.9% depending on the study, period considered and age grouping.

#### Uterine cancer

Of the nine studies including incidence trends for uterine cancer in women under 55 [8, 26, 27, 37, 39, 76–79], seven reported an increase (Table 1 and Fig. 4b).

In Canada, the rise was observed in women of all ages [37, 39], whilst in the US it was more pronounced in the under 50s, and especially in the 30–39 age group [8, 26, 27]. One study found a trend to an increase that became non-significant after adjusting for hysterectomy rates [76].

In Europe, an increase was observed in England in women 45–55-years old with a comparable increase in older women [78]. In Norway, there was a trend towards an increase in both women under and above 55 years of age, but it was significant only in the older group [77].

Compared to other countries, the largest increase was observed in New Zealand, where a study reported an APC of 9.22 (95% CI 6.10–12.50) in women <40 between 1996 and 2012, whilst the rise was smaller in older women [79].

#### Pancreatic cancer

All nine studies including trends in incidence of pancreatic cancer in patients below 60 originated from North America (Table 1 and Fig. 4c) [8, 19, 26, 37, 39, 80–83]. Taken together, these papers suggest a general increase in rates of pancreatic cancer in all age groups starting in 1940/50s with a peak in the 1970s/1980s, followed by stabilisation or decrease from 1975 onwards.

However, several studies detected a recent increase, particularly in younger adults aged under 40 [8, 26, 37, 39, 81, 82]. In Canada, the increase in younger groups was accompanied by a corresponding decrease in the over 50s [37, 39]. In two US studies, the increase in the younger age groups was specific to women, whilst in men the rise was only observed in the over 55 [26, 81]. In a Canadian study, however, the increase was more marked in younger men than women [39].

### Cancer with evidence of a decline in younger adults

#### Lung cancer

**Fig. 5:**
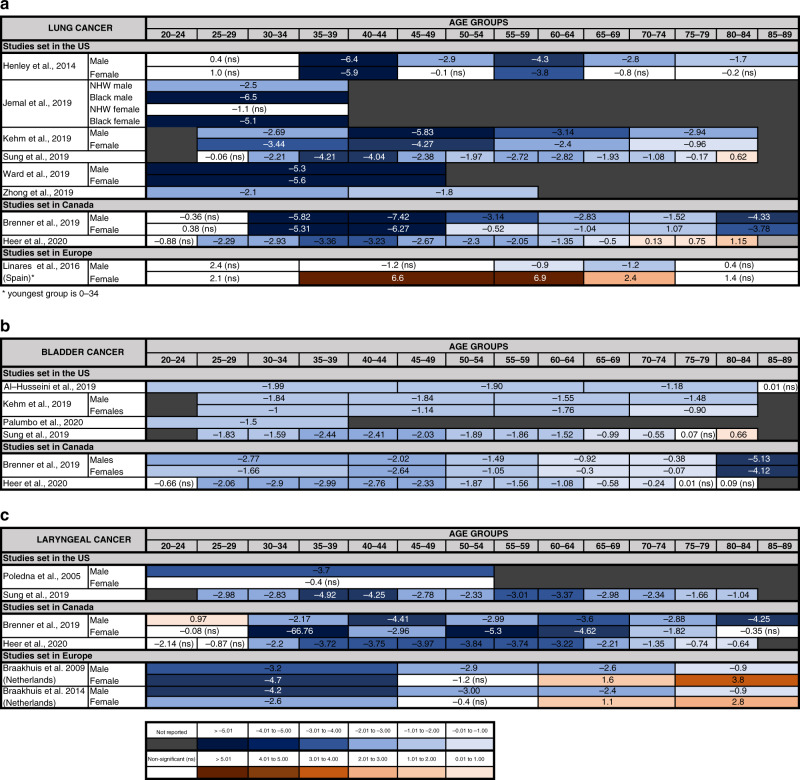
Cancers with decreasing incidence trend in younger adults. Annual percentage changes (APC) in incidence of lung (**a**), bladder (**b**) and laryngeal (**c**) cancer are reported by age group. Increases are indicated in red and decreases in blue, with darker colours corresponding to greater changes. Stable incidences are indicated in white. For simplicity, the unstratified APC is reported, when available. For studies where the unstratified APC was not available, APC is stratified by gender and/or two main ethnicities (Black and NHW, non-Hispanics White). When APC values were available for various time periods, only the most recent APC is included. For some studies, the upper age limit for the oldest group and the lowest age limit for the younger group were not reported in the original study. Detailed information of the time periods and age groups covered by the different studies is reported in Supplementary Table 2.

Of the nine studies meeting the inclusion criteria for lung cancer [8, 26, 27, 37, 39, 84–87], the majority reported a decline across all ages, but especially in under 40s, with annual changes of up to −6.5% (Table 1 and Fig. 5a). Two publications reported a small increase in older patients (>70) [8, 37]. The only discordant result was from a Spanish study, which reported no significant change in young men, but a large increase in young women [87]. When under-40s were further stratified in smaller age groups, a decrease was often reported in the older subgroups (i.e. 30–40-year olds) but not in the younger (i.e. <30-year olds). When data were stratified by gender, a more pronounced decrease was observed in men compared with women.

#### Bladder cancer

We found six studies with data on incidence for bladder cancer in the under 45s, all from North America (Table 1 and Fig. 5b) [8, 26, 37, 39, 88, 89]. After an initial rise in the 1970s/80s, a consistent decrease was observed in recent decades in all age groups. In four studies the decrease was slightly more pronounced in younger compared to older-age groups [8, 37, 39, 88]. For patients under 45, recent annual changes in incidence varied from −1.0 to –2.7%.

#### Laryngeal cancer

The six studies including data on incidence trends for laryngeal cancer in patients under 45 show an overall decrease in incidence (Table 1 and Fig. 5c) [8, 37, 39, 90–92]. In four studies, the decline was steeper in younger age groups [8, 37, 91, 92]. However, a small increase in 20–29-year olds was observed in one publication [36]. Two studies reported an increased incidence in women over 60 [91, 92].

### Cancers with the unclear trend in younger adults

#### Stomach cancer

Eleven studies reported changes in incidence in stomach cancer in patients below 55 (Table 1 and Supplementary Fig. 1a) [8, 19, 26, 37, 39, 93–98].

Six studies examined stomach cancer as a single entity, without subdivision in subtypes [19, 26, 37, 39, 96, 98]. These studies showed a decrease in older-age groups with some detecting a stable or increased rate in younger patients. APCs for people under 55 were variable, with positive and negative values reported in different studies.

Some US studies stratified stomach tumours based on the two major anatomical subtypes. In under-55s, rates of cancer of the gastric cardia were found to be stable [8, 93], whilst a trend to an increase was observed for non-cardia adenocarcinomas, with a corresponding decrease in older groups [8, 93, 95, 97].

#### Oesophageal cancer

Nine studies met inclusion criteria for oesophageal cancer (Table 1 and Supplementary Fig. 1b) [8, 19, 37, 39, 90, 93, 95, 99, 100].

Four of these studies grouped all types of oesophageal cancer types together, with contrasting results [19, 37, 39, 100]. In Canada, one study [37] observed an increase in the incidence of oesophageal cancer between 1983 and 2001, particularly in younger adults, whilst another [39] reported a decrease in under-40s. In France, a strong decrease in men aged 25–44 was found in the same time period, with a reduction of incidence in men born in the 1970s compared to those born in the 1940s [100]. A decrease in oesophageal cancer was also found in the US in all age groups, especially in adults under 50 [19].

Five studies examined trends for oesophageal squamous cell carcinoma and adenocarcinoma separately [8, 90, 93, 99]. For squamous cell carcinoma, a general decrease in incidence was observed in all studies, although in one study [99] this was only significant in over 45s, whilst in another [8] it was more pronounced in 35–44-year olds. For adenocarcinoma, two studies [93, 95] found an increase in under 50s, whilst another two [8, 99] found no significant change in younger groups.

#### Ovarian cancer

Seven papers included age-specific trends in ovarian cancer incidence in women under 50 (Table 1 and Supplementary Fig. 1c) [8, 26, 27, 37, 39, 101, 102].

One study [101] examined international trends in 27 countries and showed that incidence rates in all age groups have decreased over time in most high-income countries since the 1990s, with exception of Japan and Korea where a significant increase has been observed in older women [101, 102].

In Canada, one study [37] reported a significant decrease in women of all ages, except 20–24 and 80–84-year olds, whilst another [39] found a decline in most ages except 40–49-year olds, where the rate was stable, and 20–29-year olds where a significant increase was noticed.

In US, one publication [26] reported a significant decrease in all women above 40, but no change in the 25–39 age group. Similarly, another study [8] found a decrease in all age groups above 35, but no significant change in those under 35. However, a third study [27] found an overall significant decrease in women aged 20–49.

#### Myeloma

The four studies with data on myeloma were based in North America (Table 1 and Fig. 4d) [8, 26, 37, 39]. Both studies from the US reported an increase in all age groups but particularly under 50s, with APC increasing inversely to age [8, 26].

The two studies from Canada had contrasting results. In one study, a significant increase was observed in males aged 40–59 years and older but not 20–39-year olds and a decrease was found in 30–39-year-old women [39]. A second Canadian study reported no significant increase in those aged under 55 years but a small increased in older ages [37].

## Discussion

This is the first review examining epidemiological evidence across a range of twelve cancers with age-related referral criteria, to determine whether their incidence is increasing in younger patients. Previous reviews have generally focused on single cancers, and mostly on cancers with extensive data of an increase in young adults, such as colorectal [103]. Our findings show, importantly, that the incidence of colorectal, breast, pancreatic, kidney, and uterine cancer in younger people is rising, whilst the incidence of bladder, laryngeal and lung cancer is decreasing. Contrasting evidence was found for oesophageal, stomach and ovarian cancer and myeloma.

The reasons behind the observed trends are unclear. Changes in the prevalence of lifestyle associated risk factors in high-income countries may be contributing to the trends. Obesity, which has become more prevalent in high-income countries in the last few decades, is a risk factor for four out of the five rising cancers (colorectal, pancreas, kidney, and uterine cancer), whilst its role in early onset breast tumours is more controversial [54, 66]. Variations in patterns of childbearing and breastfeeding or increase use of oral contraception may be contributing to the rise in breast and uterine cancer incidence in younger generations [67]. Three cancers linked to smoking (lung, laryngeal and bladder) show a clear reduction of incidence, in line with the decrease in smoking rates in younger adults in recent decades. Another smoking-linked cancer, oesophageal squamous cell carcinoma, also had some evidence of decrease in younger age groups.

Changes in clinical practice, such as increased diagnostic activity, introduction of cancer screening programmes, change in management of other conditions, or change in disease classification, may have also contributed to the observed changes in incidence. Although colorectal and breast cancer screening is generally targeted to patients over 50 [104, 105], in some countries such the US screening may also be available to younger individuals, particularly those with a pertinent family history. A recent study, for example, reported that in the US up to 5% of individuals aged 40–49 had received a colonoscopy in the previous year, and that this proportion has been steadily growing from 2000 to 2015 [33]. Screening of asymptomatic individuals may result in ‘over-diagnosis’, i.e. detection of indolent tumours, which would have not progressed to become clinically significant. Therefore, an apparent increase in cancer incidence may result from increased screening. However, the increase in breast and colorectal cancer incidence is observed in individuals as young as 20–25-year old, who are unlikely to undergo screening even with a family history of the disease, and it is consistently described across a number of high-income countries worldwide, despites different health delivery systems and screening policies. In addition, some studies reported a specific increase in invasive and late-stage disease in younger age groups, rather than the rise in indolent and early-stage tumours that would be expected if the trends was solely due to increased screening.

A spike in incidence of kidney cancer in all age groups was observed in the 80s/90s due to increased imaging and incident detection of benign lesions during scans performed for other indications [72, 75]. However, a UK study looking at a range of malignancies found that overdiagnosis only partially explains the increase in kidney cancer since the 1970s [106]. The fact that the more recent rise is specific to younger patients, especially those under 40, suggests that other mechanisms may also be at play. On the contrary, the same study shows that the apparent increase in uterine cancer in the UK is likely to be driven by overdiagnosis, possibly as result of a recent change in classification guidelines [106]. A decline in use of hysterectomies for the treatment of other conditions may also have resulted in an increase in the number of uterine cancers [76].

Establishing the causes for the changes in incidence may require a detailed analysis of the mortality trends. For cancer with poor prognosis and no recent improvement in therapy, an increase in mortality is expected to follow a true increase in incidence rates, whilst increased incidence with stable or declining mortality would suggest overdiagnosis [106]. Another approach, taken by several studies included in the review, is to employ age–period-cohort modelling to disentangle trends due to factors that influence all ages (period effects), such as changes in clinical practice, those that vary by generation (cohort effects), such as exposure to risk factors, and those due to increasing age (age effects). Taken together the results of this approach suggest a real generational effect for colorectal, kidney and uterine cancers, in addition to the variations attributable to changes in clinical practice or detection of indolent tumours [8, 11, 30, 37, 38, 44, 74, 77, 78], whilst data were less clear for breast and pancreatic cancer [8, 37, 39, 54, 59, 67, 70].

### Strengths and limitations

One of the strengths of our study is the systematic approach and rigorous methodology in the literature search, selection and appraisal of evidence and data extraction. All included studies were of good quality. Most used data from large national registries, with good cover of the reported geographical area and rigorous diagnostic case ascertainment, often including microscopic confirmation. Another strength is that this study comprehensively examines and summarises incidence trends for all twelve cancers for which there are age-based criteria in the UK NICE cancer referral guidelines. Other cancers, such as melanoma, testicular and thyroid cancer are more frequently diagnosed at younger ages and therefore have no age-based criteria for referral. We focussed specifically on those cancers for which younger symptomatic patients may risk a diagnostic delay because of their age. Furthermore, our study defines ‘early on-set’ based on the specific age threshold for investigation for each cancer as reported in these clinical guidelines, rather than using an arbitrary age cut-off for all cancers (e.g., 50). Although clinical guidelines may vary between countries, NICE was chosen as it is a longstanding evidence-based national guideline with widespread adoption. The thresholds for investigation in the NICE guidelines are set at a risk of cancer of 3% or above, regardless of tumour type, resulting in different age thresholds between cancers.

One of the limitations of the study is that, to facilitate comparison, we included only publications that reported annual percentage changes in incidence. Therefore, we may have omitted some relevant studies which used other types of measures such as incidence ratios. Despite this, there was still some heterogeneity between studies, in terms of period covered, subdivision in age groups, stratification for gender and/or ethnicity or subdivision into specific anatomical, pathological or histological subtypes. Therefore, comparisons between studies need to be interpreted with caution. However, for some cancers, there was a very strong agreement between studies regarding the direction and magnitude of change. Due to this heterogeneity, meta-analysis was only possible for a subset of colorectal and breast cancer studies with similar age subdivisions. The results confirmed a significant incidence increase for both cancers in the younger age groups.

Most studies included in the review were based in North America so generalisability to other settings is uncertain, although trends were relatively consistent across countries particularly for the cancers with a larger amount of literature available. Our search used three comprehensive databases (Embase, Medline and Web of Science) but it is possible that searching different databases could have uncovered more studies from the under-represented countries, published in journals not indexed by the resources we searched.

One of the limitations of registry studies is the lack of granularity in the data. Most studies did not have information regarding patient’s risk factors, socio-economic background or comorbidities, although some US studies stratified for ethnicity. For colorectal cancers, the most consistent increase was observed in the non-Hispanic White population [14, 16, 27, 28, 32], although some studies also detected an increase in Hispanic or Black ethnicities [21–23]. For breast cancer, the most significant changes were seen in young women of Asian/Pacific Islander ethnicity [8, 48, 50, 59]. Although the evidence for stomach cancer overall was inconclusive, a significant increase in women under 50 of white ethnicity was reported in several studies [8, 93, 95, 97]. Therefore, stratification based on ethnicity and risk factors may allow to detect significant trends not apparent when younger adults are considered as a single group.

As clinical and screening guidelines group cancers based on their anatomical site, we intentionally avoided considering histological subtypes separately. However, cancer subtypes may have different aetiology and therefore distinct incidence patterns that may not become apparent when analysed as a single group. Indeed, some of the studies included in this review indicate that, whilst oesophageal squamous cell carcinoma is generally decreasing in incidence, adenocarcinoma may be increasing in under 50s. Therefore, stratification for histological subtype may reveal changes in incidence that were not apparent in our analysis.

### Clinical implications and future research

The robust evidence of an increase in colorectal cancer incidence in younger people in North America, Europe and Oceania, calls for a re-evaluation of the age threshold for the referral of symptomatic patients. This requires re-assessing the predictive value of symptoms in younger populations and a full health economic modelling to examine the balance of costs and benefits of lowering the referral thresholds. Similarly, lowering the minimum age for eligibility in colorectal cancer national screening programmes may be considered but only after careful weighting of the economic and health service implications. A modelling study in Australia has suggested that lowering bowel cancer screening threshold to 45 may be cost-effective but would increase colonoscopy demand by 3–14% and require 55–170 additional colonoscopies per additional death prevented [107]. Notably, the US Preventative Services Taskforce recently lowered their age recommendations for bowel screening from 50 to 45 years.

Whilst the increase in breast cancer incidence in young women was consistently reported across Europe and North America, the raise was modest and the overall risk remains low. For cancers with evidence of an increase, additional descriptive studies may be warranted to provide accurate estimates of current and future incidence rates in younger age groups and determine whether the overall risk is sufficiently high to justify a change in guidelines. Further research should also determine the causes behind the rise to disentangle real generational effects to those due to changes in clinical practice, increase testing and overdiagnosis. Policy changes regarding referral of symptomatic patients or screening could be considered in those countries and for those cancers with a confirmed increasing trend. However, despite increases in incidence, the low absolute risk in young people may mean that a change in age thresholds may not be cost-effective or justifiable. Nevertheless, family doctors should be aware of these trends and that the possibility of cancer should not be dismissed solely based on age. Mechanisms should be in place to allow clinicians to take further action if they suspect serious disease in younger patients.

Clarifying the role of preventable causes will help underpin more effective population health policies aimed at cancer prevention. More research is also needed to establish whether the rising incidence trend in the younger population is associated to specific subgroups, to allow improved risk stratification and facilitate more targeted interventions. Furthermore, lowering of referral or screening age may be facilitated by the development of new highly sensitive and specific testing modalities.

In conclusion, our study shows that the incidence of colorectal, breast, kidney, pancreatic and uterine cancer is increasing in younger people, whilst lung, laryngeal and bladder cancers are becoming less common. Policymakers need to be aware of these trends when reviewing guidelines and screening programmes. Addressing preventable risk factors such as excess weight may help curb the increase of these malignancies in younger generations.

## Supplementary information

Supplementary legends

Supplementary Figure 1

Supplementary Figure 2

Supplementary Figure 3

Supplementary Table 1

Supplementary Table 2

Supplementary Table 3

Supplementary methods

## Data Availability

As the study is a review, data are already publicly available. A spreadsheet containing the data extracted from the 98 studies included in the review is available from the corresponding author on request.
