# Supplementary material for: Incidence trends for twelve cancers in younger adults—a rapid review
Source: Br J Cancer. 2022 Feb 7;126(10):1374–86. doi: 10.1038/s41416-022-01704-x (PMC9090760; doi:10.1038/s41416-022-01704-x)
Supplement: Supplementary file 1 — Supplementary legends [file 41416_2022_1704_MOESM1_ESM.docx]

**Supplementary Legends**

**Figures**

**Suppl. Figure 1.** **Recent trends in the incidence of stomach (A), oesophageal (B) ovarian (C) cancer and myeloma (D).** Annual percentage changes (APC) in incidence are reported by age group. Increases are indicated in red and decreases in blue, with darker colours corresponding to greater changes. Stable incidences are indicated in white. For simplicity, the unstratified APC is reported, when available. For studies where the unstratified APC was not available, APC is stratified by gender and/or two main ethnicities (Black and NHW, Non-Hispanics White). When APC values where available for various time periods, only the most recent APC is included. For some studies, the upper age limit for the oldest group and the lowest age limit for the younger group were not reported in the original study. Detailed information of the time periods and age groups covered by the different studies is reported in Supplementary Table 2.

**Suppl. Figure 2.** A. Forest plot from meta-analysis of published incidence trends for colorectal cancer in patients <50 years; B. Forest plot from meta-analysis of published incidence trends for colorectal cancer in patients <40 years; C. Forest plot from meta-analysis of published incidence trends for colorectal cancer in patients 20-29, 30-39 and 40-49 years-old. Effect size is measured as annual percentage change (APC). (*) indicates studies that did not report full confidence intervals or p-values.

**Suppl. Figure 3.** Forest plot from meta-analysis of published incidence trends for breast cancer in women <50 years. Studies were pooled based on age subdivision. Effect size is measured as annual percentage change (APC). (*) indicates studies that did not report full confidence intervals or p-values.

**Tables**

**Supplementary Table 1. Age-specific limits for urgent cancer referral in adults, based on the UK cancer recognition and referral guidelines.** For each cancer, only the guidelines with a specific age-threshold are reported. These guidelines, and their age cut-off, were used to define early on-set cancer for the purposes of this review. ‘Younger’ patients were defined as those aged under the age-cut off for each specific cancer.

**Supplementary Table 2. Detailed information regarding the 98 studies included in the review.** Cancer type, country, quality score based on the Joanna Briggs appraisal tool for prevalence studies, cancer register from which the data was derived, age subdivision, type of outcome measured and years covered by the analysis is reported for all studies. For studies where multiple APC were reported for different time periods, the most recent time-period was considered for the review and is indicated in column J. For some studies APC covered different periods depending on age groups. This is indicated as 'variable' and readers should refer to the original study for more details.

**Supplementary Table 3. Pooled estimate of the annual percent change (APC) in incidence by age group for breast and colorectal cancers.** In bold, age group showing significant changes in incidence.
