# Supplementary material for: Incidence trends for twelve cancers in younger adults—a rapid review
Source: Br J Cancer. 2022 Feb 7;126(10):1374–86. doi: 10.1038/s41416-022-01704-x (PMC9090760; doi:10.1038/s41416-022-01704-x)
Supplement: Supplementary file 2 — Supplementary Figure 1 [file 41416_2022_1704_MOESM2_ESM.pdf]

Supplementary Figure 1

not reported	> -5.01	-4.01 to -5.00	-3.01 to -4.00	-2.01 to -3.00	-1.01 to -2.00	-0.01 to -1.00
non significant (ns)	> 5.01	4.01 to 5.00	3.01 to 4.00	2.01 to 3.00	1.01 to 2.00	0.01 to 1.00

A.

<table>
<tr><th colspan="2" rowspan="2">STOMACH CANCER</th><th colspan="14">AGE GROUP</th></tr>
<tr><th>20-24</th><th>25-29</th><th>30-34</th><th>35-39</th><th>40-44</th><th>45-49</th><th>50-54</th><th>55-59</th><th>60-64</th><th>65-69</th><th>70-74</th><th>75-79</th><th>80-84</th><th>85-89</th></tr>
<tr><td colspan="16">Studies set in the US</td></tr>
<tr><td colspan="2">Hussan et al., 2020</td><td colspan="6">0.7</td><td colspan="3">-0.5 (ns)</td><td colspan="2">-1.1</td><td colspan="3">-1.8</td></tr>
<tr><td rowspan="2">Kehm et al., 2019</td><td>male</td><td rowspan="2"></td><td colspan="3">0.20 (ns)</td><td colspan="3">-0.06 (ns)</td><td colspan="3">-1.71</td><td colspan="3">-1.77</td><td rowspan="2"></td></tr>
<tr><td>female</td><td colspan="3">1.98</td><td colspan="3">1.16</td><td colspan="3">-1.31</td><td colspan="3">-1.64</td></tr>
<tr><td colspan="2">Merchant et al., 2017</td><td colspan="6">0.33 (ns)</td><td colspan="3">-2.28</td><td colspan="5">-2.56</td></tr>
<tr><td colspan="16">Non-cardia adenocarcinoma</td></tr>
<tr><td rowspan="2">Anderson et al., 2010</td><td>GNCA White</td><td rowspan="2"></td><td colspan="3">2.7</td><td colspan="4">-0.8</td><td colspan="5">-1.8</td><td rowspan="2"></td></tr>
<tr><td>GNCA Black</td><td colspan="3">-1.7 (ns)</td><td colspan="4">-2.1</td><td colspan="5">-1.4</td></tr>
<tr><td>Anderson et al., 2018</td><td>GNCA NHW</td><td colspan="6">1.3</td><td colspan="8">-2.6</td></tr>
<tr><td rowspan="2">Islami et al., 2019</td><td>GNCA Male</td><td colspan="4">1.3</td><td colspan="2">0 (ns)</td><td colspan="2">0.8 (ns)</td><td colspan="2">-2.5</td><td colspan="4">-2.8</td></tr>
<tr><td>GNCA Female</td><td colspan="4">2.9</td><td colspan="2">2.5</td><td colspan="2">0.8</td><td colspan="2">-1.5</td><td colspan="4">-2.5</td></tr>
<tr><td>Sung et al., 2019</td><td>GNCA</td><td></td><td>1.6</td><td>2.16</td><td>1.7</td><td>1.77</td><td>1.14</td><td>0.58</td><td>-0.75</td><td>-1.53</td><td>-1.73</td><td>-1.63</td><td>-1.82</td><td>-2.09</td><td></td></tr>
<tr><td>Wang et al., 2020</td><td>GNCA NHW</td><td colspan="6">1.24</td><td colspan="8">-2.28</td></tr>
<tr><td colspan="16">Adenocarcinoma of the gastric cardia</td></tr>
<tr><td rowspan="2">Islami et al., 2019</td><td>GCA Male</td><td colspan="4">0.3 (ns)</td><td colspan="2">-0.1 (ns)</td><td colspan="2">2.3</td><td colspan="2">1.0</td><td colspan="4">0.3</td></tr>
<tr><td>GCA Female</td><td colspan="4">0.3 (ns)</td><td colspan="2">1.9</td><td colspan="2">0.5 (ns)</td><td colspan="2">0.6 (ns)</td><td colspan="4">0.4 (ns)</td></tr>
<tr><td>Sung et al., 2019</td><td>GCA</td><td></td><td>0.05 (ns)</td><td>0.94 (ns)</td><td>0.13 (ns)</td><td>-015 (ns)</td><td>-0.22 (ns)</td><td>-0.45</td><td>-0.64</td><td>-0.52</td><td>-0.19 (ns)</td><td>0.10 (ns)</td><td>0.30 (ns)</td><td>0.91</td><td></td></tr>
<tr><td colspan="16">Studies set in Canada</td></tr>
<tr><td rowspan="2">Brenner et al., 2020</td><td>male</td><td colspan="4">-0.42 (ns)</td><td colspan="2">0.84 (ns)</td><td colspan="2">3.05</td><td colspan="2">-2.37</td><td colspan="2">-1.82</td><td colspan="2">-0.56 (ns)</td></tr>
<tr><td>female</td><td colspan="4">-0.8</td><td colspan="2">0.23 (ns)</td><td colspan="2">0.83 (ns)</td><td colspan="2">9.74 (ns)</td><td colspan="2">-2.46</td><td colspan="2">-9.23</td></tr>
<tr><td colspan="2">Heer et al., 2020</td><td>2.03</td><td>0.51 (ns)</td><td>0.24 (ns)</td><td>0.01 (ns)</td><td>-0.71</td><td>-1.49</td><td>-2.05</td><td>-2.24</td><td>-2.25</td><td>-2.21</td><td>-2.10</td><td>-2.17</td><td>-2.50</td><td></td></tr>
<tr><td colspan="16">Studies set in Asia</td></tr>
<tr><td rowspan="2">Song et al., 2015 (S. Korea)</td><td>male</td><td colspan="4">-3.7</td><td colspan="3">0.2 (ns)</td><td colspan="3">-0.1 (ns)</td><td colspan="2">-0.5 (ns)</td><td colspan="2" rowspan="2"></td></tr>
<tr><td>female</td><td colspan="4">-0.8 (ns)</td><td colspan="3">1.7</td><td colspan="3">-1.1</td><td colspan="2">-1.0</td></tr>
</table>

GNCA: Gastric Non-Cardia Adenocarcinoma; GCA: Adenocacinoma of the Gastric Cardia

B.

<table>
<tr><th colspan="2" rowspan="2">OESOPHAGEAL CANCER</th><th colspan="14">AGE GROUP</th></tr>
<tr><th>20-24</th><th>25-29</th><th>30-34</th><th>35-39</th><th>40-44</th><th>45-49</th><th>50-54</th><th>55-59</th><th>60-64</th><th>65-69</th><th>70-74</th><th>75-79</th><th>80-84</th><th>85-89</th></tr>
<tr><td colspan="16">Studies set in the US</td></tr>
<tr><td colspan="2">Hussan et al., 2020</td><td colspan="6">-1.8</td><td colspan="3">-1.1</td><td colspan="2">-1.2</td><td colspan="3">-0.7 (ns)</td></tr>
<tr><td colspan="16">Adenocarcinoma</td></tr>
<tr><td>Anderson et al., 2018</td><td>OAC NHW</td><td colspan="6">1.3</td><td colspan="8">1.9</td></tr>
<tr><td rowspan="2">Islami et al., 2019</td><td>OAC Male</td><td colspan="4">0.9 (ns)</td><td colspan="2">-1.0 (ns)</td><td colspan="2">-1.0</td><td colspan="2">-1.4</td><td colspan="4">-0.4 (ns)</td></tr>
<tr><td>OAC Female</td><td colspan="4"></td><td colspan="2">2.4</td><td colspan="2">1.3</td><td colspan="2">0.5 (ns)</td><td colspan="4">1.4 (ns)</td></tr>
<tr><td>Sung et al., 2019</td><td>OAC</td><td></td><td>1.90 (ns)</td><td>1.42 (ns)</td><td>0.61 (ns)</td><td>0.38 (ns)</td><td>0.17 (ns)</td><td>0.3</td><td>0.93</td><td>1.44</td><td>1.47</td><td>1.45</td><td>1.77</td><td>2.28</td><td></td></tr>
<tr><td>Trivers et al., 2008</td><td>OAC</td><td colspan="5">2.7 (ns)</td><td colspan="3">1.8 (ns)</td><td colspan="3">1.9</td><td colspan="3">2.7</td></tr>
<tr><td colspan="16">Squamous cell carcinoma</td></tr>
<tr><td>Sung et al., 2019</td><td>OSSC</td><td colspan="2"></td><td>-1.96 (ns)</td><td>-4.84</td><td>-4.49</td><td>-3.46</td><td>-3.1</td><td>-3.73</td><td>-4.37</td><td>-3.99</td><td>-3.42</td><td>-2.44</td><td>-1.9</td><td></td></tr>
<tr><td>Trivers et al., 2009</td><td>OSSC</td><td colspan="5">-3.4(ns)</td><td colspan="3">-3.7</td><td colspan="3">-4.1</td><td colspan="3">-2.6</td></tr>
<tr><td rowspan="2">Polednak et al., 2005</td><td>OSSC male</td><td colspan="7">-3.9</td><td colspan="7" rowspan="2"></td></tr>
<tr><td>OSSC female</td><td colspan="7">-5.2</td></tr>
<tr><td rowspan="2">Islami et al., 2019</td><td>OSSC Male</td><td colspan="4" rowspan="2"></td><td colspan="2">-4.7</td><td colspan="2">-2.0</td><td colspan="2">-3.8</td><td colspan="4">-3.0</td></tr>
<tr><td>OSSC Female</td><td colspan="2">-2.5</td><td colspan="2">2.1 (ns)</td><td colspan="2">0 (ns)</td><td colspan="4">-2.5</td></tr>
<tr><td colspan="16">Studies set in Canada</td></tr>
<tr><td colspan="2">Heer et al., 2020</td><td>0.20 (ns)</td><td>2.57 (ns)</td><td>2.27</td><td>1.63</td><td>1.32</td><td>0.99</td><td>0.54</td><td>0.40</td><td>0.26</td><td>0.38</td><td>0.44</td><td>0.72</td><td>0.70</td><td></td></tr>
<tr><td rowspan="2">Brenner et al., 2020</td><td>male</td><td colspan="2">-0.59</td><td colspan="2">-0.21 (ns)</td><td colspan="2">0.74</td><td colspan="2">0.59</td><td colspan="2">0.53</td><td colspan="2">0.53</td><td colspan="2">-6.06 (ns)</td></tr>
<tr><td>female</td><td colspan="2">-1.27</td><td colspan="2">-0.93</td><td colspan="2">-0.47 9ns)</td><td colspan="2">-0.26 (ns)</td><td colspan="2">-0.34</td><td colspan="2">-0.42</td><td colspan="2">-6.31</td></tr>
<tr><td colspan="16">Studies set in Europe</td></tr>
<tr><td>Gilhodes et al., 2015</td><td>men</td><td colspan="4">-6.24</td><td colspan="10"></td></tr>
</table>

OAC: oesophageal Adenocarcinoma; OSSC: Oeasophageal Squamous Cell Carcinoma

C.

<table>
<tr><th colspan="2" rowspan="2">OVARIAN CANCER *</th><th colspan="14">AGE GROUPS</th></tr>
<tr><th>20-24</th><th>25-29</th><th>30-34</th><th>35-39</th><th>40-44</th><th>45-49</th><th>50-54</th><th>55-59</th><th>60-64</th><th>65-69</th><th>70-74</th><th>75-79</th><th>80-84</th><th>85-89</th></tr>
<tr><td colspan="16">Studies set in the US</td></tr>
<tr><td>Kehm et al., 2019</td><td></td><td rowspan="2"></td><td colspan="3">0.04 (ns)</td><td colspan="3">-0.87</td><td colspan="3">-2.01</td><td colspan="3">-1.35</td><td rowspan="2"></td></tr>
<tr><td colspan="2">Sung et al., 2019</td><td>-0.26 (ns)</td><td>-0.54 (ns)</td><td>-1.01</td><td>-1.35</td><td>-1.63</td><td>-1.94</td><td>-2.18</td><td>-2.2</td><td>-1.91</td><td>-1.87</td><td>-1.85</td><td>-1.4</td></tr>
<tr><td colspan="2">Ward et al., 2019</td><td colspan="6">-0.7</td><td colspan="8"></td></tr>
<tr><td colspan="16">Studies set in Canada</td></tr>
<tr><td colspan="2">Brenner et al., 2020</td><td colspan="2">2.31</td><td colspan="2">-1.21</td><td colspan="2">0.7 (ns)</td><td colspan="2">-1.15</td><td colspan="2">-1.04</td><td colspan="2">-0.68</td><td colspan="2">-7.53</td></tr>
<tr><td colspan="2">Heer et al., 2020</td><td>0.11 (ns)</td><td>-0.8</td><td>-0.6</td><td>-0.91</td><td>-0.72</td><td>-0.97</td><td>-1.14</td><td>-1.23</td><td>-1.19</td><td>-1.05</td><td>-0.86</td><td>-0.58</td><td>-0.26 (ns)</td><td></td></tr>
<tr><td colspan="16">Studies set in Asia</td></tr>
<tr><td colspan="2">Kim et al., 2016 (S. Korea)</td><td colspan="4">1.87</td><td colspan="4">2.48</td><td colspan="6">3.02</td></tr>
</table>
