# Supplementary figures and images for: Incidence trends for twelve cancers in younger adults—a rapid review
Source: Br J Cancer. 2022 Feb 7;126(10):1374–86. doi: 10.1038/s41416-022-01704-x (PMC9090760; doi:10.1038/s41416-022-01704-x)

## Supplementary Figure 2

A.

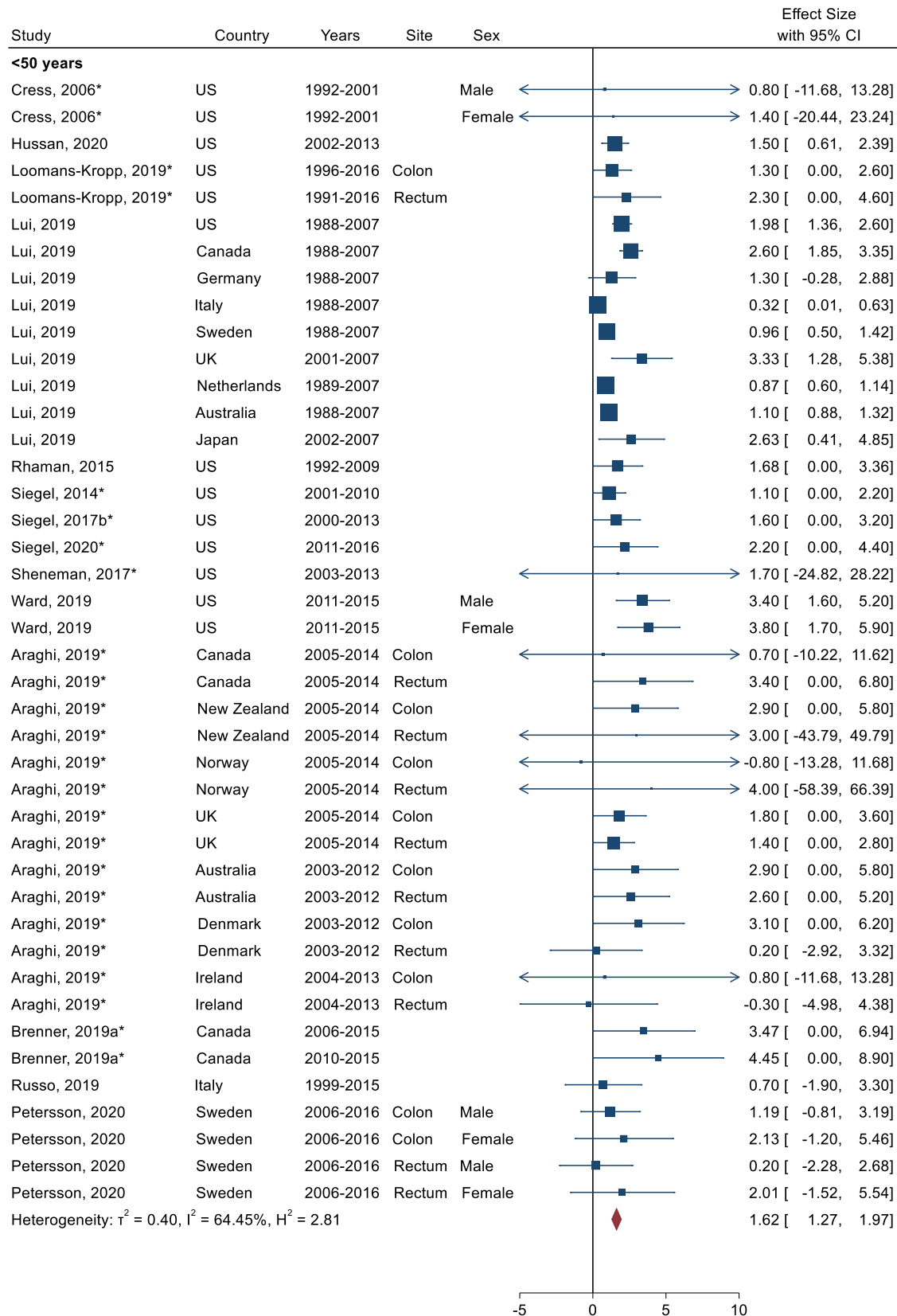

B.

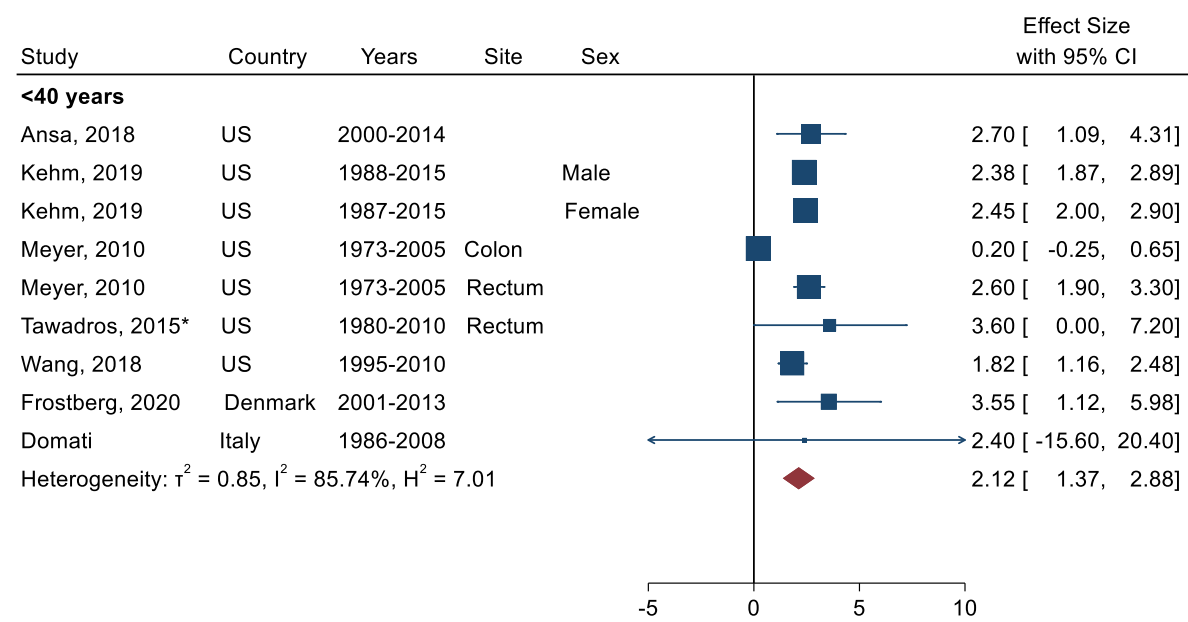

C.

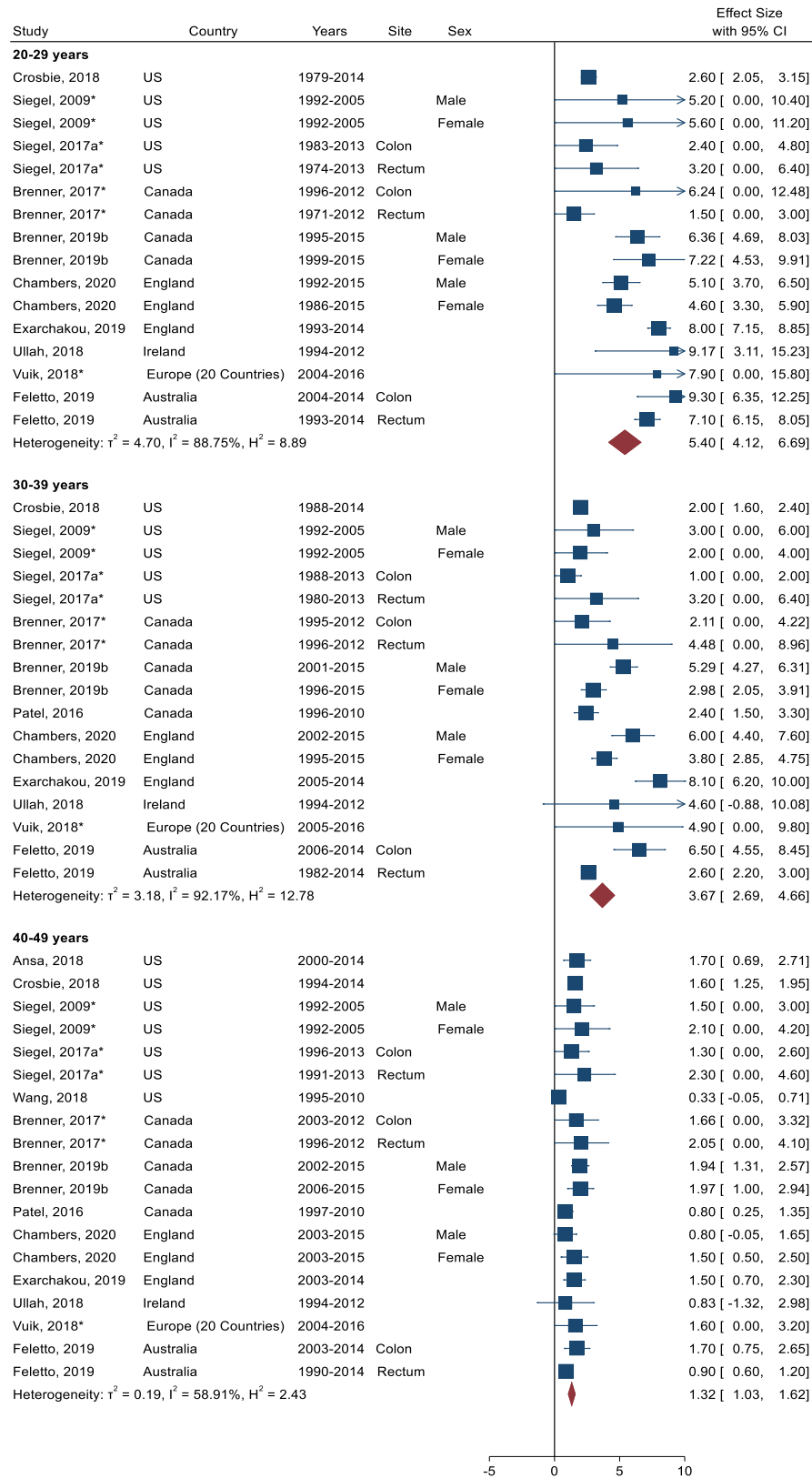

Supplement: Supplementary file 3 — Supplementary Figure 2 [file 41416_2022_1704_MOESM3_ESM.pdf]

## Supplementary Figure 3

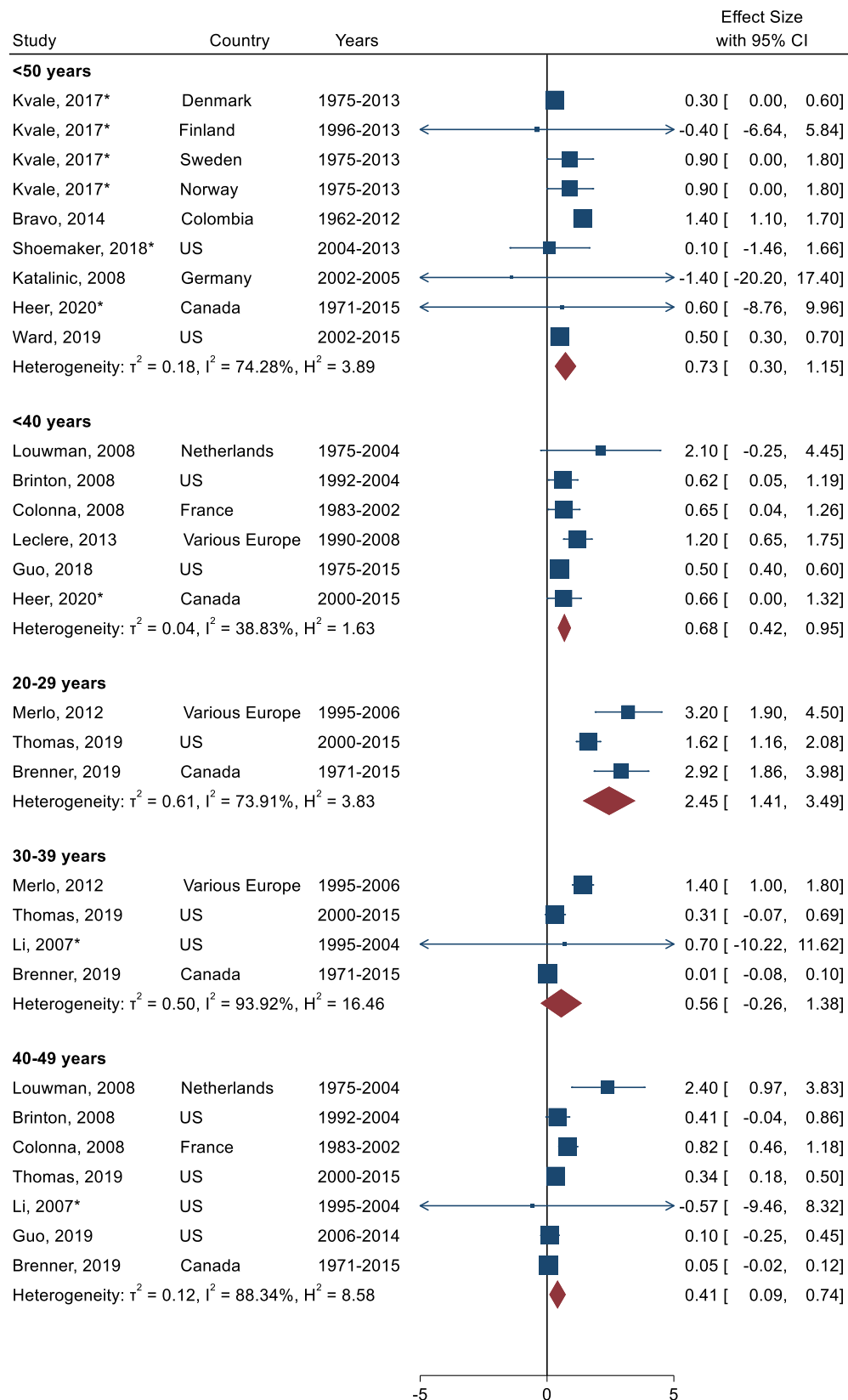

Random-effects REML model

Supplement: Supplementary file 4 — Supplementary Figure 3 [file 41416_2022_1704_MOESM4_ESM.pdf]
