# Supplementary material for: Incidence trends for twelve cancers in younger adults—a rapid review
Source: Br J Cancer. 2022 Feb 7;126(10):1374–86. doi: 10.1038/s41416-022-01704-x (PMC9090760; doi:10.1038/s41416-022-01704-x)
Supplement: Supplementary file 5 — Supplementary Table 1 [file 41416_2022_1704_MOESM5_ESM.docx]

**Supplementary Table 1**

| **Type of cancer** | **Guideline** | **Age cut-off** |
| --- | --- | --- |
| **Colorectal** | Urgent referral recommended for patients:   - aged 40 and over with unexplained weight loss and abdominal pain; - aged 50 and over with unexplained rectal bleeding; - aged 60 and over with: iron-deficiency anaemia or changes in their bowel habit, or tests show occult blood in their faeces.   Urgent referral to be considered for patients:   - under 50 with rectal bleeding and any of the following unexplained symptoms or findings: abdominal pain, change in bowel habit, weight loss, iron-deficiency anaemia. | 50 |
| **Bladder** | Urgent referral recommended for patients:   - aged 45 and over with unexplained visible haematuria without urinary tract infection or visible haematuria that persists or recurs after successful treatment of urinary tract infection; - aged 60 and over with unexplained non-visible haematuria and either dysuria or a raised white cell count on a blood test. | 45 |
| **Lung** | Urgent referral recommended for patients:   - aged 40 and over with unexplained haemoptysis;   Urgent chest X-ray for patients:   - aged 40 and over if they have 2 or more of the following unexplained symptoms; or if they have ever smoked and have 1 or more of the following unexplained symptoms: cough, suspected fatigue, shortness of breath, chest pain, weight loss, appetite loss;   Urgent chest X-ray to be considered for patients:   - aged 40 and over with any of the following: persistent or recurrent chest infection, finger clubbing, supraclavicular lymphadenopathy or persistent cervical lymphadenopathy, chest signs consistent with lung cancer, thrombocytosis. | 40 |
| **Oesophagu**s | Urgent upper gastrointestinal endoscopy recommended for patients:   - aged 55 and over with weight loss and any of the following: upper abdominal pain, reflux, dyspepsia;   Non-urgent direct access to upper gastrointestinal endoscopy to be considered for patients:   - aged 55 or over with treatment-resistant dyspepsia or upper abdominal pain with low haemoglobin levels, or raised platelet count with any of the following: nausea vomiting weight loss reflux dyspepsia upper abdominal pain, or nausea or vomiting with any of the following: weight loss, reflux dyspepsia, upper abdominal pain. | 55 |
| **Pancreas** | Urgent referral recommended for patients:   - aged 40 and over who have jaundice   Urgent direct access to CT or ultrasound scan for patients:   - aged 60 and over with weight loss and any of the following: diarrhoea, back pain, abdominal pain nausea, vomiting, constipation, new-onset diabetes. | 60 |
| **Stomach** | Urgent direct access upper gastrointestinal endoscopy recommended for patients:   - aged 55 and over with weight loss and any of the following: upper abdominal pain, reflux, dyspepsia;   Non-urgent direct access upper gastrointestinal endoscopy to be considered for patients:   - aged 55 or over with: treatment-resistant dyspepsia or upper abdominal pain with low haemoglobin levels or raised platelet count and any of the following: nausea, vomiting, weight loss, reflux, dyspepsia, upper abdominal pain, or nausea or vomiting | 55 |
| **Breast** | Urgent referral recommended for patients:   - aged 30 and over with an unexplained breast lump with or without pain; - aged 50 and over with any of the following symptoms in one nipple only: discharge, retraction, other changes of concern.   Urgent referral to be considered for patients:   - aged 30 and over with an unexplained lump in the axilla.   Non-urgent referral to be considered for patients:   - aged under 30 with an unexplained breast lump with or without pain. | 50 |
| **Ovarian** | Appropriate tests for ovarian cancer recommended in any woman:   - 50 or over who has experienced symptoms within the last 12 months that suggest irritable bowel syndrome (IBS) for the first time | 50 |
| **Endometrial** | Urgent referral recommended for patients:   - aged 55 and over with post-menopausal bleeding;   Urgent referral to be considered for patients:   - aged under 55 with post-menopausal bleeding;   Direct access to ultrasound scan to be considered for patients:   - aged 55 and over with: unexplained symptoms of vaginal discharge who are presenting with these symptoms for the first time or have thrombocytosis or report haematuria; visible haematuria and low haemoglobin levels or thrombocytosis or high blood glucose levels. | 55 |
| **Renal** | Urgent referral recommended for patients:   - aged 45 and over who have: unexplained visible haematuria without urinary tract infection or visible haematuria that persists or recurs after successful treatment of urinary tract infection. | 45 |
| **Laryngeal** | Urgent referral to be considered for patients:   - aged 45 and over with persistent unexplained hoarseness or an unexplained lump in the neck | 45 |
| **Myeloma** | Offer a full blood count, blood tests for calcium and plasma viscosity or erythrocyte sedimentation rate to patients:   - aged 60 and over with persistent bone pain, particularly back pain, or unexplained fracture   Offer very urgent protein electrophoresis and a Bence-Jones protein urine test to patients:   - aged 60 and over with hypercalcaemia or leukopenia and a presentation that is consistent with possible myeloma | 60 |
