# Supplementary material for: Incidence trends for twelve cancers in younger adults—a rapid review
Source: Br J Cancer. 2022 Feb 7;126(10):1374–86. doi: 10.1038/s41416-022-01704-x (PMC9090760; doi:10.1038/s41416-022-01704-x)
Supplement: Supplementary file 6 — Supplementary Table 2 [file 41416_2022_1704_MOESM6_ESM.docx]

| **Cancer type** | **Authors** | **Title** | **Country** | **Quality score** | **Cancer register** | **Age subdivision** | **Outcome** | **Years covered** | **Period considered for APC** |
| --- | --- | --- | --- | --- | --- | --- | --- | --- | --- |
| bladder | Al-Husseini et al. | Trends in the incidence and mortality of transitional cell carcinoma of the bladder for the last four decades in the USA: a SEERbased analysis | US | 9 | SEER 9 | 20-44, 45-64, 65-84, >84 | APC | 1974-2014 | variable |
| bladder | Palumbo et al. | Bladder cancer incidence rates and trends in young adults aged 20-39 years | US | 9 | SEER 18 | 20-39, 40+ | AAPC | 2000-2016 | - |
| breast | Katalinik et al. | Decline in breast cancer incidence after decrease in utilisation of hormone replacement therapy | Germany | 7 | Cancer registry of Schleswig-Holstein | <50, 50-69, 70+ | APC | 2001-2005 | - |
| breast | Kvale et al. | Prostate and breast cancer in four Nordic countries: A comparison of incidence and mortality trends across countries and age groups 1975-2013 | North Europe | 7 | NORDCAN | <50, 50–69, 70–79, 80+ | EAPC | 1975-2013 | variable |
| breast | Pollan et al. | Recent Changes in Breast Cancer Incidence in Spain, 1980 – 2004 | Spain | 7 | 16 spanish cancer registries | 25-44, 45-64, 65+ | APC | 1980-2004 | <45 1980-2004, 45-64 2001-2004, 65+ 1995-2004 |
| breast | Glass et al. | Breast cancer incidence, 1980 – 2006: combined roles of menopausal hormone therapy, screening mammography, and estrogen receptor status | US | 7 | Kaiser Permanente Northwest | <45, 45-59, 60+ | APC | 1980-2006 | 1980-2006 for under 40, 2000-2006 for 45-59 and 2001-2006 for 60+ |
| breast | Bravo et al. | Burden of breast cancer in Cali, Colombia: 1962-2012 | Colombia | 7 | Cali Cancer Registry | <50, 50-69, 70+ | APC | 1962-2012 | - |
| breast | Bouchardy et al. | Changing pattern of age-specific breast cancer incidence in the Swiss canton of Geneva | Switzerland | 7 | Geneva Cancer Registry | 25-49, 50-69, 70+ | APC | 1975-2006 | 2002-2006 50-66, 1975-2006 for 25-49 and 70+ |
| breast | DeSantis et al. | Trends in stage at diagnosis for young breast cancer patients in the United States. | US | 7 | NAACCR | 20-39 | APC | 2001-2015 | - |
| breast | Heer et al. | The incidence of breast cancer in Canada 1971-2015: trends in screening-eligible and young-onset age groups | Canada | 8 | Canadian Cancer Registry | 20-49, 50-74, <40 | AAPC | 1971-2015 | 2000-2015 for under 50 and 2004-2015 over 50 |
| breast | Guo et al. | Breast Cancer Incidence by Stage Before and After Change in Screening Guidelines | US | 8 | NPCR and SEER | 40-49, 50-74 | APC | 2006-2014 | - |
| breast | Hou and Huo | A trend analysis of breast cancer incidence rates in the United States from 2000 to 2009 shows a recent increase | US | 8 | SEER 18 | 20–39, 40–49, 50–69, 70+ | APC | 2000-2009 | variable |
| breast | Li and Daling | Changes in breast cancer incidence rates in the United States by histologic subtype and race/ethnicity, 1995 to 2004 | US | 8 | SEER 13 | 30-39, 40-49, 50-59, 60-69, 70-79, 80+ | APC | 1995-2004 | variable |
| breast | Smigal et al. | Trends in breast cancer by race and ethnicity: update 2006 | US | 8 | NAACRR and SEER | <50, >50 | APC | 1975-2002 | variable |
| breast | Colonna et al. | Is breast cancer incidence increasing among young women? An analysis of the trend in France for the period 1983-2002 | France | 8 | 7 French registers | 15-39, 40-49, 50-74, 75+ | APC | 1983-2002 | - |
| breast | Leclère B et al. | Trends in incidence of breast cancer among women under 40 in seven European countries: a GRELL cooperative study | Europe | 9 | 7 European cancer registries | 15-35, 35-39 | AAPC | 1990-2008 | - |
| breast | Merlo et al. | Breast cancer incidence trends in European women aged 20-39 years at diagnosis | Europe | 9 | 17 European cancer registries | 20-29, 30-39 | AIC | 1995-2006 | - |
| breast | Louwman et al. | On the rising trends of incidence and prognosis for breast cancer patients diagnosed 1975-2004: a long-term population-based study in southeastern Netherlands | Netherlands | 9 | Eindhoven Cancer Registry | <40, 40-49, 50-69, 70+ | EACP | 1975-2004 | 1995-2004 |
| breast | Guo et al. | Trends in breast cancer mortality by stage at diagnosis among young women in the United States | US | 9 | SEER9 | 20-39 | APC | 1975-2015 | 1994-2015 |
| breast | Johnson et al. | Incidence of breast cancer with distant involvement among women in the United States, 1976 to 2009 | US | 9 | SEER 9, SEER 13, SEER 18 | 25-39, 40-54, 55-69, 70-84 | APC | 1976-2009 | 2000-2009 (SEER18 only data) |
| breast | Shoemaker et al. | Differences in breast cancer incidence among young women aged 20-49 years by stage and tumor characteristics, age, race, and ethnicity, 2004-2013 | US | 9 | NPCR and SEER | 20-34, 35-39, 40-44, 45-49 | APC | 2004-2013 | - |
| breast | Thomas et al. | Incidence and Survival Among Young Women With Stage I-III Breast Cancer: SEER 2000-2015 | US | 9 | SEER 18 | 20-29, 30-39, 40-49 | AAPC | 2000-2015 | - |
| breast | Forjaz de Lacerda et al. | Breast cancer in Portugal: Temporal trends and age-specific incidence by geographic regions | Portugal | 9 | Portuguese Cancer Registry | 30-44, 45-69, 70-84 | APC | 1998-2011 | - |
| breast | Brinton et al. | Recent trends in breast cancer among younger women in the United States | US | 9 | SEER 13 | <40, 40-49, 50+ | APC | 1992-2004 | - |
| breast | Aarts et al. | Reduction of socioeconomic inequality in cancer incidence in the South of the Netherlands during 1996-2008 | Netherlands | 9 | Eindhoven cancer registry | 24-44, 45-64, 65+ | APC | 1996-2008 | - |
| colorectal | Ansa et al. | Evaluation of colorectal Cancer incidence trends in the United States (2000-2014). | US | 9 | SEER 18 | <40, 40-49, 50-59, 60-69, 70-79, >80 | APC | 2000-2014 | - |
| colorectal | Araghi et al. | Changes in colorectal cancer incidence in seven high-income countries: a population-based study | Multiple | 8 | Various national registries | 20-29, 30-39, 40-49 (<50), 51-74, 75+ | AAPC/APC | variable-2014 | - |
| colorectal | Augustus et al. | Is increased colorectal screening effective in preventing distant disease? | US | 8 | SEER 18 | <50, 50-65, 65+ | APC | 2000-2014 | 2000-2014 for <50; variable for >50 |
| colorectal | Austin et al. | Changes in colorectal cancer incidence rates in young and older adults in the United States: what does it tell us about screening | US | 8 | SEER and NPCR | <50, 50+ | APC | 1998-2009 | - |
| colorectal | Bailey et al. | Increasing disparities in the age-related incidences of colon and rectal cancers in the United States, 1975-2010 | US | 9 | SEER 9 | 20-34, 35-49, 50-74, 75+ | APC | 1975-2010 | - |
| colorectal | Brenner et al. | National Trends in Colorectal Cancer Incidence Among Older and Younger Adults in Canada | Canada | 8 | National Cancer Incidence Reporting System (1969 - 1992) Canadian Cancer Registry (1992-2015) | <50, >50 | APC | 1969-2015 | variable |
| colorectal | Brenner et al. | Increasing colorectal cancer incidence trends among younger adults in Canada | Canada | 8 | National Cancer Incidence Reporting System (1969 - 1992) Canadian Cancer Registry (1992-2015) | 20–29, 30–39, 40–49, 50–54, 55–59,60–64, 65–69, 70–74, 75–79, 80–84, 85+ | APC | 1971-2012 | variable |
| colorectal | Chambers et al. | Demographic trends in the incidence of young-onset colorectal cancer: a population-based study | England | 8 | National Cancer Registration and Analysis Service | 20-29, 30-39, 40-49 | APC | 1974-2015 | variable |
| colorectal | Cress et al. | Secular changes in colorectal cancer incidence by subsite, stage at diagnosis, and race/ethnicity, 1992-2001. | US | 8 | SEER 12 | 0-49, 50-64, 65+ | EAPC | 1992-2001 | - |
| colorectal | Crosbie et al. | Trends in colorectal cancer incidence among younger adults-Disparities by age, sex, race, ethnicity, and subsite | US | 8 | New Jersey State Cancer Register | 20-29, 30-39, 40-49, 50-59, 60-69, 70-79, 80+ | APC | 1979-2014 | variable |
| colorectal | Domati et al. | Incidence, clinical features and possible etiology of early onset (<=40 years) colorectal neoplasms | Italy | 8 | Colorectal Cancer Registry of Modena | <=40, >40 | EAPC | 1984-2008 | 1986-2008 |
| colorectal | Exarchakou et al. | Colorectal cancer incidence among young adults in England: Trends by anatomical sub-site and deprivation | England | 9 | National Cancer Registry for England | 20–29, 30–39, 40–49, 50–59,60–69, 70–79, 80–99 | APC | 1971-2014 | variable |
| colorectal | Fedewa et al. | Are temporal trends in colonoscopy among young adults concordant with colorectal cancer incidence? | US | 8 | SEER 18 | 40-44, 45-49, 50-54 | APC | 2000-2015 | - |
| colorectal | Feletto et al. | Trends in Colon and Rectal Cancer Incidence in Australia from 1982 to 2014: Analysis of Data on Over 375,000 Cases | Australia | 8 | Australian Cancer Incidence and Mortality | 20-29, 30-39, 40-49, 50-54, 55-59, 60-64, 65-69, 70-74, 75-79, 80-84, 85+ | APC | 1982-2014 | variable |
| colorectal | Frostberg and Rahr | Clinical characteristics and a rising incidence of early-onset colorectal cancer in a nationwide cohort of 521 patients aged 18-40 years | Denmark | 8.5 | Danish Cancer registry / Danish Colorectal Cancer Group (DCCG) | <40 | APC | 2001-2013 | - |
| colorectal | Giddings et al. | Going against the tide: increasing incidence of colorectal cancer among Koreans, Filipinos,and south | US | 8 | California Cancer Registry | <50, >50 | APC | 1988-2007 | 1988-2007, except South Asian 1991-2007 |
| colorectal | Loomans-Kropp and Umar | Increasing Incidence of Colorectal Cancer in Young Adults | US | 8 | SEER 9 and SEER 18 | <50, >50 | APC | 1980-2016 | variable |
| colorectal | Lu et al. | International incidence trends in early- and late-onset colorectal cancer: a population-based study | Multiple | 7 | CI5C | <50, >50 | EAPC | 1988-2012 | - |
| colorectal | Lui et al. | Global Increasing Incidence of Young-Onset Colorectal Cancer Across 5 Continents: A Joinpoint Regression Analysis of 1,922,167 Cases | Multiple | 7 | CI5C Plus | <50, >50 | APC | 1988-2007 | - |
| colorectal | Meyer et al. | Increasing incidence of rectal cancer in patients aged younger than 40 years: An analysis of the surveillance, epidemiology, and end results database | US | 9 | SEER | <40 | APC | 1973-2005 | - |
| colorectal | Patel and De | Trends in colorectal cancer incidence and related lifestyle risk factors in 15-49-year-olds in Canada, 1969-2010 | Canada | 7 | Canadian Cancer Registry | 15-29, 30-39, 40-49 | APC | 1969-2010 | 1996/7-2010 |
| colorectal | Petersson et al. | Increasing incidence of colorectal cancer among the younger population in Sweden | Sweden | 7 | Swedish Cancer Registry | <50, 50-74, >75 | AAPC | 1970-2016 | 2006-2016 |
| colorectal | Rhaman et al. | Increased risk for colorectal cancer under age 50 in racial and ethnic minorities living in the United States | US | 7 | SEER 13 | <50, >50 | AAPC | 1992-2009 | - |
| colorectal | Russo et al. | Increased incidence of colon cancer among individuals younger than 50 years: A 17 years analysis from the cancer registry of the municipality of Milan, Italy | Italy | 9 | Cancer Registry for Milan | <50, 50-64, >65 | APC | 1999-2015 | 1999-2015 except age 65+ 2007-2015 |
| colorectal | Sheneman et al. | The impact of young adult colorectal cancer: Incidence and trends in Colorado | US | 9 | Colorado central cancer registry | 0-49, >50 | EAPC | 1992-2013 | 2003-2013 |
| colorectal | Siegel et al. | Colorectal cancer statistics, 2014 | US | 8 | SEER 9, SEER13 and NPCR | 0-49, 50-64, 65+ | APC | 2001-2010 | 2001-2010 for <50; 2008-2010 others |
| colorectal | Siegel et al. | Colorectal Cancer Incidence Patterns in the United States, 1974-2013 | US | 9 | SEER 9 | 20-29, 30-39, 40-49, 50-54, 55-59, 60-64, 65-69, 70-74, 75-79, 80-84, 85+ | APC | 1974-2013 | variable |
| colorectal | Siegel et al. | Increase in Incidence of Colorectal Cancer Among Young Men and Women in the United States | US | 9 | SEER 13 | 20-29, 30-39, 40-49 | APC | 1992-2005 | - |
| colorectal | Siegel et al. | Colorectal cancer statistics, 2017 | US | 8 | SEER 9, SEER 13 and NPCR | 0-49, 50-64, 65+ | APC | 2000-2013 | 2000-2013 except 65+ 2008-2013 |
| colorectal | Siegel et al. | Colorectal cancer statistics, 2020 | US | 8 | SEER 9, SEER 13 and NPCR | 0-49, 50-64, 65+ | APC | 1995-2016 | 2011-2016 |
| colorectal | Siegel et al. | Global patterns and trends in colorectal cancer incidence in young adults | Multiple | 8 | CI5C and IACR | <50, >50 | AAPC | 2008-2012 | - |
| colorectal | Tawadros et al. | Adenocarcinoma of the rectum in patients under age 40 is increasing: impact of signet-ring cell histology | US | 9 | SEER 9 and SEER 18 | 20-39, >40 | APC | 1980-2010 | - |
| colorectal | Troeung et al. | Increasing Incidence of Colorectal Cancer in Adolescents and Young Adults Aged 15-39 Years in Western Australia 1982-2007: Examination of Colonoscopy History | Australia | 7 | Western Australia Cancer Registry | 15-19, 20-24, 25-29, 30-34, 35-39 | APC | 1982-2007 | - |
| colorectal | Ullah et al. | Changing trends in age and stage of colorectal cancer presentation in Ireland - From the nineties to noughties and beyond | Ireland | 9 | Irish National Cancer Registry | 20-29, 30-39, 40-49, 50-59, 60-69, 70-79, 80+ | APC/AAPC | 1994-2012 | - |
| colorectal | Van Beck et al. | Colorectal Cancer Incidence and Mortality Rates Among New York City Adults Ages 20-54 years during 1976-2015 | US | 9 | New York State Cancer Registry | 20-49, 50-54 | APC | 1976-2015 | age 20-49: 1990-2015; age 50-54: 1993-2015 |
| colorectal | Vuik et al. | Increasing incidence of colorectal cancer in young adults in Europe over the last 25 years | Europe | 9 | European Cancer Registries | 20-29, 30-39, 40-49 | APC | 1990-2016 | 2004/5-2016 |
| colorectal | Wang et al. | Rising Incidence of Colorectal Cancer Among Young Hispanics in Texas | US | 9 | Texas Cancer Registry | 20-39, 40-49, 50-74, 75+ | APC | 1995-2010 | - |
| uterine | Duncan et al. | Cancer of the body of the uterus: trends in mortality and incidence in England, 1985–2008. | England | 7 | Oxford Cancer Intelligence Unit | 45-49, 50-54, 55-59, 60-64, 65-69, 70-74, 75-79, 80-84, 85+ | AAPC | 1985-2008 | 2001-2008 |
| uterine | Scott et al. | Rapid increase in endometrial cancer incidence and ethnic differences in New Zealand | New Zealand | 8 | New Zealand Cancer Registry | <40, 40-49, 50-74,75+ | APC | 1996-2012 | - |
| uterine | Lindemann et al. | Endometrial cancer incidence trends in Norway during 1953-2007 and predictions for 2008-2027 | Norway | 9 | Norvegian Cancer Registry | <55, 55-79 | APC | 1953-2007 | 1998-2007 |
| uterine | Temkin et al. | Hysterectomy-corrected rates of endometrial cancer among women younger than age 50 in the United States | US | 9 | SEER 12 | 20-29, 30-34, 35-39, 40-44, 45-49 | APC | 1992-2010 | - |
| oesophageal | Trivers et al. | Trends in esophageal cancer incidence by histology, United States, 1998-2003 | US | 9 | NPCR and SEER | <45, 45-59, 60-74, 75+ | APC | 1998-2003 | - |
| gastric | Wang et al. | Increasing Incidence of Advanced Non-cardia Gastric Cancers Among Younger Hispanics in the USA | US | 8 | NPCR and SEER | <50, >50 | AAPC | 2001-2014 | - |
| gastric | Song et al. | Age and sex interactions in gastric cancer incidence and mortality trends in Korea | Korea | 9 | Cancer Registry of Korea | 20-39, 40-54, 65-69, 70-79 | APC | 1999-2010 | - |
| gastric and oeophageal | Anderson et al. | The Changing Face of Noncardia Gastric Cancer Incidence Among US Non-Hispanic Whites. | US | 9 | NAACCR | <50, >50 | EAPC | 1995-2013 | - |
| gastric | Anderson et al. | Age-specific trends in incidence of noncardia gastric cancer in US adults | US | 9 | SEER 9, SEER 13 and SEER 17 | 25-39, 40-59, 60-84 | EAPC | 1977-2006 | - |
| gastric | Merchant et al. | A rising trend in the incidence of advanced gastric cancer in young Hispanic men | US | 9 | SEER 13 | 20-49, 50-64, >65 | APC | 1992-2011 | - |
| gastric and oeophageal | Islami et al. | Incidence Trends of Esophageal and Gastric Cancer Subtypes by Race, Ethnicity, and Age in the United States, 1997-2014 | US | 9 | NAACCR | 0-39, 40-49, 50-59, 60-69,>70 | EAPC | 1997-2014 | 2010-2014 |
| Kidney | De P et al. | Trends in incidence, mortality, and survival for kidney cancer in Canada, 1986–2007. | Canada | 8 | National Cancer Incidence and Reporting System and Canadian Cancer Registry | 15-44, 45-54, 55-64, 65-74, 75+ | APC | 1986-2010 | 1986-2007, except females 75+ 1992-2007 |
| kidney | King et al. | Continued increase in incidence of renal cell carcinoma, especially in young patients and high grade disease: United States 2001 to 2010 | US | 9 | NPCR and SEER | 20-24, 25-29, 30-34, 35-39, 40-44, 45-49, 50-54, 55-59, 60-64, 65-69, 70-74, 75-79, 80-84, 85+ | APC | 2001-2010 | - |
| kidney | Nepple et al. | Population based analysis of the increasing incidence of kidney cancer in the United States: evaluation of age specific trends from 1975 to 2006 | US | 9 | SEER 9 | 25-39, 41-49, 50-59, 60-69, 70-79, 80+ | APC | 1975-2006 | 1991-2006 |
| kidney | Palumbo et al. | Renal cell carcinoma incidence rates and trends in young adults aged 20-39 years | US | 9 | SEER 18 | 20-39 | AAPC | 2000-2016 | - |
| kidney | Tyson et al. | Age-period-cohort analysis of renal cell carcinoma in United States adults | US | 9 | SEER 9 | 25-39, 40-59, 60-84 | APC | 1973-2008 | - |
| laryngeal | Braakhuis et al. | Incidence and survival trends of head and neck squamous cell carcinoma in the Netherlands between 1989 and 2011 | Netherlands | 8 | Netherland cancer registry | <45, >45 | EAPC | 1989-2011 | - |
| laryngeal | Braakhuis et al. | Oral and oropharyngeal cancer in The Netherlands between 1989 and 2006: Increasing incidence, but not in young adults. | Netherlands | 8 | Netherland cancer registry | <45, 45-59, 60-74, 75+ | EAPC | 1989-2006 | - |
| lung | Jemal et al. | The convergence of lung cancer rates between blacks and whites under the age of 40, United States | US | 6 | SEER 12 | 20-39 | APC | 1992-2006 | - |
| lung | Henley et al. | Lung cancer incidence trends among men and women--United States, 2005-2009 | US | 7 | CDC national prgram of cancer registries and SEER. | <35, 35-44, 45-54, 55-64, 65-74, 75+ | APC | 2005-2009 | - |
| lung | Linares et al. | Trends in lung cancer incidence by histologic subtype in the south of Spain, 1985-2012: a population-based study | Spain | 8 | Granada Cancer Registry | 0-34, 35-54, 55-64, 65-74, 75+ | APC | 1985-2012 | 1985-2012 except male 65-74 1994-2012 |
| lung | Zhong et al. | Trends and Patterns of Disparities in Burden of Lung Cancer in the United States, 1974-2015 | US | 9 | SEER 9 | 20-39, 40-59, 60-79, 80+ | AAPC | 1974-2015 | - |
| ovarian | Kim et al. | Incidence of epithelial ovarian cancer according to histologic subtypes in Korea, 1999 to 2012 | Korea | 7 | Korea Central Cancer Registry | <40, 40-59, 60+ | APC | 1999-2012 | - |
| ovarian | Cabasag et al. | The influence of birth cohort and calendar period on global trends in ovarian cancer incidence | Multiple | 8 | CI5C | 25-49, 50-74 | AAPC | 1998-2012 | variable |
| pancreas | Gad et al. | Temporal trends of pancreatic ductal adenocarcinoma in young adults in the United States: A Population-Based Study | US | 8 | SEER 18 | 25-34, 35-39 | APC | 2000-2017 | 2000-2014 |
| pancreas | Tavakkoli et al. | Racial Disparities and Trends in Pancreatic Cancer Incidence and Mortality in the United States | US | 8 | NPCR and SEER | 30-39, 40-49, 50-59, 60-69, 70-79, 80-89 | APC | 2001-2015 | variable |
| pancreas | Gordon-Dseagu et al. | Pancreatic cancer incidence trends: evidence from the Surveillance, Epidemiology and End Results (SEER) population-based data | US | 9 | SEER 9, SEER 13, SEER 18 | 25-34, 35-44, 45-54, 55-64, 65-74, 75-84, 85+ | APC | 1992-2013 | - |
| pancreas | Zhang et al. | Trends in pancreatic cancer incidence in nine SEER Cancer Registries, 1973-2002 | US | 9 | SEER 9 | <60, >60 | EAPC | 1973-2002 | 1994-2000 for <60, 1983-2002 for >60 |
| various | Gilhodes et al. | Incidence of major smoking-related cancers: trends among adults aged 20-44 in France from 1982 to 2012 | France | 7 | 6 French Cancer Registries | 20-44 | APC | 1982-2012 | - |
| various (oesophagus and laryngeal) | Polednak et al. | Recent trends in incidence rates for selected alcohol-related cancers in the United States | US | 7 | SEER 12 | 20-54 | APC | 1992-2001 | - |
| various | Hussan et al. | Rising Incidence of Colorectal Cancer in Young Adults Corresponds With Increasing Surgical Resections in Obese Patients | US | 7.5 | SEER | 20–49, 50–64, 65–74, and 74+ | AAPC | 2002-2013 | - |
| various | Heer et al. | Emerging cancer incidence trends in Canada: The growing burden of young adult cancers | Canada | 8.5 | CI5plus | 20-24, 25-29, 30-34, 35-39, 40-44, 45-49, 50-54, 55-59, 60-64, 65-69, 70-74, 75-79, 80-84 | AAPC | 1983-2012 | - |
| various | Ward et al. | Annual Report to the Nation on the Status of Cancer, Featuring Cancer in Men and Women Age 20-49 Years | US | 8.5 | NAACCR | 20-49 | APC | 1999-2015 | - |
| various | Brenner et al. | Age-standardized cancer-incidence trends in Canada, 1971-2015 | Canada | 9 | Canadian Cancer Registry | 20-29, 30-39, 40-49, 50-59, 60-69, 70-79, 80-89 | APC | 1971-2015 | variable |
| various | Kehm et al. | 40 Years of Change in Age- and Stage-Specific Cancer Incidence Rates in US Women and Men | US | 9 | SEER9 | 25-39, 40-54, 55-69, 70-84 | APC | 1975-2015 | variable |
| various | Sung et al. | Emerging cancer trends among young adults in the USA: analysis of a population-based cancer registry | US | 9 | NAACCR | 25-29, 30-34, 35-39, 40-44, 45-49, 50-54, 55-59, 60-64, 65-69, 70-74, 75-79, 80-84 | AAPC | 1995-2014 | - |

**Supplementary Table 2**
