# Supplementary material for: Incidence trends for twelve cancers in younger adults—a rapid review
Source: Br J Cancer. 2022 Feb 7;126(10):1374–86. doi: 10.1038/s41416-022-01704-x (PMC9090760; doi:10.1038/s41416-022-01704-x)
Supplement: Supplementary file 7 — Supplementary Table 3 [file 41416_2022_1704_MOESM7_ESM.docx]

**Supplementary Table 3**

|  | **Breast** | | | | | | **Colorectal** | | | | | |
| --- | --- | --- | --- | --- | --- | --- | --- | --- | --- | --- | --- | --- |
|  | **All studies** | | | **Studies that reported confidence intervals or full p-values only** | | | **All studies** | | | **Studies that reported confidence intervals or full p-values only** | | |
| **Age group** | **N** | **Pooled APC**  **(95% CI)** | **I^2^** | **N** | **Pooled APC**  **(95% CI)** | **I^2^** | **N** | **Pooled APC (95% CI)** | **I^2^** | **N** | **Pooled APC (95% CI)** | **I^2^** |
| <50 | 9 | **0.73**  **(0.30, 1.15)** | 74% | 3 | **0.94**  **(0.06, 1.82)** | 92% | 42 | **1.62**  **(1.27, 1.97)** | 64% | 17 | **1.57**  **(1.08, 2.06)** | 86% |
| <40 | 6 | **0.68**  **(0.42, 0.95)** | 39% | 5 | **0.71**  **(0.39, 1.02)** | 48% | 9 | **2.12**  **(1.37, 2.88)** | 86% | 8 | **2.07**  **(1.30, 2.85)** | 87% |
| 20-29 | 3 | **2.45**  **(1.41, 3.49)** | 74% | 3 | **2.45**  **(1.41, 3.49)** | 74% | 16 | **5.40**  **(4.12, 6.69)** | 89% | 9 | **6.24**  **(4.79, 7.69)** | 91% |
| 30-39 | 4 | 0.56  (-0.26, 1.38) | 94% | 3 | 0.56  (-0.27, 1.38) | 96% | 17 | **3.67**  **(2.69, 4.66)** | 92% | 10 | **4.27**  **(2.98, 5.56)** | 95% |
| 40-49 | 7 | **0.41**  **(0.09, 0.74)** | 88% | 6 | **0.42**  **(0.09, 0.74)** | 90% | 19 | **1.32**  **(1.03, 1.62)** | 59% | 12 | **1.26**  **(0.91, 1.60)** | 72% |

N = number of estimates pooled (many studies reported more than one group of interest i.e. different countries or sex or site)

I^2^ = Heterogeneity I^2^
