# Supplementary material for: Incidence trends for twelve cancers in younger adults—a rapid review
Source: Br J Cancer. 2022 Feb 7;126(10):1374–86. doi: 10.1038/s41416-022-01704-x (PMC9090760; doi:10.1038/s41416-022-01704-x)
Supplement: Supplementary file 8 — Supplementary methods [file 41416_2022_1704_MOESM8_ESM.docx]

# Search Strategy

**Embase Classic+Embase (Ovid) 1947 to 2020 August 20**

**Search date: 21-08-2020**

Search Strategy:

--------------------------------------------------------------------------------

1 cancer incidence/ (75785)

2 incidence/ (416525)

3 prevalence/ (730872)

4 1 or 2 or 3 (1164320)

5 age distribution/ (143908)

6 onset age/ (84228)

7 5 or 6 [Age issues] (225268)

8 exp *colorectal tumor/ep [Epidemiology] (1205)

9 exp *colon tumor/ep (8031)

10 exp *rectum tumor/ep (7032)

11 exp *bladder tumor/ep (1894)

12 exp *lung tumor/ep [Epidemiology] (7458)

13 exp *esophagus tumor/di [Diagnosis] (9060)

14 exp *pancreas tumor/ep [Epidemiology] (1804)

15 exp *stomach tumor/ep [Epidemiology] (3904)

16 exp *breast tumor/ep [Epidemiology] (14597)

17 exp *ovary tumor/ep [Epidemiology] (2818)

18 exp *endometrium tumor/ep [Epidemiology] (1257)

19 exp *kidney tumor/ep [Epidemiology] (1662)

20 exp *larynx tumor/ep [Epidemiology] (857)

21 *myeloma/ep (38)

22 *multiple myeloma/ep (609)

23 or/8-22 [Epidemiology of Cancers with age threshold] (52017)

24 exp cohort analysis/ (606799)

25 exp longitudinal study/ (143914)

26 exp prospective study/ (623478)

27 exp follow up/ (1614141)

28 cohort*.tw. (1019604)

29 or/24-28 [BMJ Evidence Filter] (2944578)

30 register/ (116017)

31 cancer registry/ (35774)

32 29 or 30 or 31 (3037772)

33 4 and 7 and 23 and 32 (561)

34 (juvenile/ or exp aged/) not (adult/ or middle aged/ or young adult/) (992012)

35 33 not 34 [EmTree search limited to Adults] (548)

36 ((young* adult* or age*) adj7 incidence* adj7 (cancer* or neoplas* or tumo* or carcinoma* or adenocarcinoma* or metasta* or malignan* or oncolog*)).tw,kw. (10314)

37 ((young* adult* or age*) adj7 prevalen* adj7 (cancer* or neoplas* or tumo* or carcinoma* or adenocarcinoma* or metasta* or malignan* or oncolog*)).tw,kw. (1770)

38 ((young* adult* or age*) adj7 (rate or rates) adj7 (cancer* or neoplas* or tumo* or carcinoma* or adenocarcinoma* or metasta* or malignan* or oncolog*)).tw,kw. (9413)

39 ((young* adult* or age*) adj7 trend* adj7 (cancer* or neoplas* or tumo* or carcinoma* or adenocarcinoma* or metasta* or malignan* or oncolog*)).tw,kw. (1401)

40 or/36-39 [Cancer incidence and Age issues textwords] (18838)

41 (colon or colorectal or rectal or rectum or bladder or lung? or pulmonary or oesophag* or esophag*).tw,kw. (2512956)

42 (pancrea* or stomach or gastric or breast? or ovary or ovari* or endometr* or renal or kidney? or laryngeal*).tw,kw. (3161815)

43 41 or 42 [Cancers with Age Threshold free text words] (5294622)

44 (adult? or young* men or young* women or young* people or middle-aged or "early onset" or "young onset").tw,kw. (1861584)

45 40 and 43 and 44 [Incidence of Cancer with Age threshold limited to Adults_ text word search] (1511)

46 35 or 45 (2038)

47 exp erratum/ (207918)

48 editorial/ or letter/ or note/ (2481809)

49 case report/ (2611782)

50 (comment* or letter? or editorial? or note? or "case report*" or "expert opinion").ti. (710139)

51 48 or 49 or 50 (4984722)

52 51 not 47 [Editorials or case reports but not retractions] (4979314)

53 45 not 52 (1461)

54 limit 53 to english language (1375)

55 limit 54 to yr="1995 -Current" (1236)

56 limit 55 to conference abstracts (438)

57 55 not 56 (798)

**Ovid MEDLINE(R) and Epub Ahead of Print, In-Process & Other Non-Indexed Citations and Daily 1946 to August 19, 2020**

**Search date: 20-08-2020**

Search Strategy:

--------------------------------------------------------------------------------

1 incidence/ (263277)

2 prevalence/ (292732)

3 1 or 2 (531321)

4 age distribution/ (66371)

5 exp *age factors/ (10577)

6 4 or 5 [Age issues] (76613)

7 exp *Colorectal Neoplasms/ep (5859)

8 *Urinary Bladder Neoplasms/ep (1216)

9 exp *Lung Neoplasms/ep (5014)

10 exp *Esophageal Neoplasms/ep (1624)

11 exp *Pancreatic Neoplasms/ep (1122)

12 exp *Stomach Neoplasms/ep (2381)

13 exp *Breast Neoplasms/ep (9713)

14 exp *Ovarian Neoplasms/ep (1613)

15 exp *Endometrial Neoplasms/ep (814)

16 exp *Kidney Neoplasms/ep (1025)

17 *Laryngeal Neoplasms/ep (584)

18 exp *Multiple Myeloma/ep (469)

19 or/7-18 [Epidemiology of Cancers with age threshold] (29858)

20 exp Cohort Studies/ (2021121)

21 exp Registries/ (96766)

22 20 or 21 [cohort or registry study research] (2079499)

23 3 and 19 and 6 and 22 [MeSH Incidence Cancer_Epidemiology Age_Issues Cohorts_Registries] (712)

24 (adolescent/ or exp aged/ or exp child/ or exp infant/) not (adult/ or middle aged/ or young adult/) (2539902)

25 23 not 24 [MeSH search limited to Adults] (690)

26 ((young* adult* or age*) adj7 incidence* adj7 (cancer* or neoplas* or tumo* or carcinoma* or adenocarcinoma* or metasta* or malignan* or oncolog*)).tw,kw. (6995)

27 ((young* adult* or age*) adj7 prevalen* adj7 (cancer* or neoplas* or tumo* or carcinoma* or adenocarcinoma* or metasta* or malignan* or oncolog*)).tw,kw. (1058)

28 ((young* adult* or age*) adj7 (rate or rates) adj7 (cancer* or neoplas* or tumo* or carcinoma* or adenocarcinoma* or metasta* or malignan* or oncolog*)).tw,kw. (6546)

29 ((young* adult* or age*) adj7 trend* adj7 (cancer* or neoplas* or tumo* or carcinoma* or adenocarcinoma* or metasta* or malignan* or oncolog*)).tw,kw. (1035)

30 26 or 27 or 28 or 29 [Cancer incidence and Age issues textwords] (12587)

31 (colon or colorectal or rectal or rectum or bladder or lung? or pulmonary or oesophag* or esophag*).tw,kw. (1655095)

32 (pancrea* or stomach or gastric or breast? or ovary or ovari* or endometr* or renal or kidney? or laryngeal*).tw,kw. (2186071)

33 31 or 32 [Cancers with Age Threshold free text words] (3617996)

34 (adult? or young* men or young* women or young* people or middle-aged or "early onset" or "young onset").tw,kw. (1347612)

35 30 and 33 and 34 [Incidence of Cancer with Age threshold limited to Adults_ text word search] (946)

36 25 or 35 (1615)

37 (increas* or decreas* or declin* or rise? or rising).tw,kw. (6958060)

38 36 and 37 [Increasing Cancer Incidence Age_Issues Adults] (1095)

39 Published Erratum/ or Retraction of Publication/ (90231)

40 letter/ or editorial/ or news/ or newspaper article/ or Comment/ (2080486)

41 case reports/ (2117233)

42 (comment* or letter? or editorial? or note? or "case report*" or "expert opinion").ti. (506213)

43 40 or 41 or 42 (4164795)

44 43 not 39 [Editorials or case reports but not retractions] (4162319)

45 38 not 44 (1075)

46 limit 45 to english language (1016)

47 limit 46 to yr="1995 -Current" (933)

**Web of Science databases:**

- Science Citation Index-Expanded (Web of Science) 1900-present
- Social Sciences Citation Index (Web of Science) 1900-present
- Emerging Sources Citation Index (Web of Science) 2015-present

**Search date: 20-08-2020**

# 13 657 #11 not #12 . Limited to Timespan=1995-2020

# 12 529,919 TITLE: (comment* or letter? or editorial? or note? or "case report*" or "expert opinion")

# 11 702 #10 AND #9 AND #8 AND #5

# 10 10,006,090 TOPIC: (increas* or decreas* or declin* or rise* or rising)

# 9 1,841,316 TS=(adult* or young* men or young* women or young* people or middle-aged or "early onset" or "young onset")

# 8 3,018,944 #7 OR #6

# 7 1,783,430 TOPIC: (pancrea* or stomach or gastric or breast? or ovary or ovari* or endometr* or renal or kidney? or laryngeal*)

# 6 1,388,468 TOPIC: (colon or colorectal or rectal or rectum or bladder or lung? or pulmonary or oesophag* or esophag*)

# 5 11,574 #4 OR #3 OR #2 OR #1

# 4 1,114 TOPIC: (("young* adult*" or age*) near/7 trend* near/7 (cancer* or neoplas* or tumo* or carcinoma* or adenocarcinoma* or metasta* or malignan* or oncolog*) )

# 3 5,945 TOPIC: (("young* adult*" or age*) near/7 (rate or rates) near/7 (cancer* or neoplas* or tumo* or carcinoma* or adenocarcinoma* or metasta* or malignan* or oncolog*) )

# 2 6,224 TOPIC: ((("young* adult*" or age*) near/7 incidence* near/7 (cancer* or neoplas* or tumo* or carcinoma* or adenocarcinoma* or metasta* or malignan* or oncolog*) ))

# 1 1,125 TOPIC: (("young* adult*" or age*) near/7 prevalen* near/7 (cancer* or neoplas* or tumo* or carcinoma* or adenocarcinoma* or metasta* or malignan* or oncolog*) )

# Data Extraction

1. Author and date
2. Country
3. Type of study
4. Age subdivision and number of patients per group
5. Years covered by data
6. Source of data
7. Outcome (e.g. APC, AAPC, EAPC)
8. Change in incidence (yes/no plus details - e.g APC and 95% CI for each age group)
9. Characteristic of cases if known (e.g. ethnicity, socio-economic, BMI)
10. Quality score (based on the Joanna Briggs tool)
11. Other notes (e.g. histological subtypes included)

# Meta-analysis

Random-effects meta-analysis were used to derive pooled estimates of the APCs and forest plots used for graphical visualisation of results. Pooled estimates were calculated for studies reporting trends for similar age groups if there were at least three studies per age group. Due to the wide variation in the time periods included in the studies we were unable to stratify by time-period. The standard errors of the APC were derived using reverse Z-tests on the reported confidence intervals or reported p-values. When full p-values were not reported and only APC indicated as statistically significant (usually p<0.05) then the p-value was assumed to be equal to the value (e.g. p=0.05 for p<0.05). For trends only reported as not significant (e.g. p>0.05 or p=NS) we estimated the confidence interval assuming it crosses 0 with a high p-value. Sensitivity analysis was conducted restricted to studies that reported confidence intervals and full p-values only. Pooled estimates were calculated for breast and colorectal cancers for age groups <50 years. If studies reported trends for 20-49 this was classified as <50 years, whilst 15-39 and 20-39-year groups were classified as <40 years. Age groups included <50-years, <40-years and 20-29, 30-39 and 40-49 years.
